# Gut-derived metabolites influence neurodevelopmental gene expression and Wnt signaling events in a germ-free zebrafish model

**DOI:** 10.1186/s40168-022-01302-2

**Published:** 2022-08-23

**Authors:** Victoria Rea, Ian Bell, Taylor Ball, Terence Van Raay

**Affiliations:** Department of Molecular and Cellular Biology, University of Guelph, Guelph, Canada

**Keywords:** Germ-free, Wnt, Neurodevelopment, Metabolites, Microbiome, Zebrafish

## Abstract

**Background:**

Small molecule metabolites produced by the microbiome are known to be neuroactive and are capable of directly impacting the brain and central nervous system, yet there is little data on the contribution of these metabolites to the earliest stages of neural development and neural gene expression. Here, we explore the impact of deriving zebrafish embryos in the absence of microbes on early neural development as well as investigate whether any potential changes can be rescued with treatment of metabolites derived from the zebrafish gut microbiota.

**Results:**

Overall, we did not observe any gross morphological changes between treatments but did observe a significant decrease in neural gene expression in embryos raised germ-free, which was rescued with the addition of zebrafish metabolites. Specifically, we identified 354 genes significantly downregulated in germ-free embryos compared to conventionally raised embryos via RNA-Seq analysis. Of these, 42 were rescued with a single treatment of zebrafish gut-derived metabolites to germ-free embryos. Gene ontology analysis revealed that these genes are involved in prominent neurodevelopmental pathways including transcriptional regulation and Wnt signaling. Consistent with the ontology analysis, we found alterations in the development of Wnt dependent events which was rescued in the germ-free embryos treated with metabolites.

**Conclusions:**

These findings demonstrate that gut-derived metabolites are in part responsible for regulating critical signaling pathways in the brain, especially during neural development.

Video abstract

**Supplementary Information:**

The online version contains supplementary material available at 10.1186/s40168-022-01302-2.

## Introduction

Animals and microbes share a deep evolutionary history as animal development emerged and co-evolved with a microbe-rich environment [1]. The gut microbiome codes for biochemical functions that host genomes cannot encode, such as the breakdown of otherwise indigestible macromolecules into products that their hosts can utilize [2]. The microbiome has been implicated in neural development and function, and consequently, perturbation of the microbiota is implicated in neurological disease [3–6]. It is known that metabolites act as communication signals between host and microbiome in the form of neuromodulators or neurotransmitters [7]. Both neural and circulatory routes have been proposed as a means of gut-brain signaling including the vagus nerve and enteric nervous system (ENS) and direct absorption from the intestinal lumen into the blood stream [8, 9]. The vagus nerve and ENS are sensitive to gamma amino butyric acid (GABA), serotonin, histamine, and acetylcholine, all of which are produced by the gut microbiota [9]. Small molecules such as short-chain fatty acids (SCFAs) produced by the gut microbiota can enter the blood stream via the intestinal lumen and cross the blood-brain barrier (BBB) where they can then interact with the brain and affect neural transmission [10]. Therefore, the correlation between the gut microbiome and the brain is unlikely due solely to the presence of bacteria, but more likely due to the metabolites and small molecules that bacteria produce as fermentation by-products. Recent studies have shown that metabolites alone can affect neural development. For example, SCFAs have been shown to reduce the inflammatory response of cultured human cells modeling microglial immune functions [11]. Further, Yang et al. (2020) found that the growth rate of human neural progenitor cells is affected by treatment with SCFAs such that physiologically relevant doses increase the growth rate, but high levels of SCFAs have toxic effects on these cells. The researchers also show that SCFA treatment affects the expression of neurogenesis genes [12]. SCFAs have also been shown to modulate microglia in a germ-free, Alzheimer’s disease mouse model [13], and intraventricular infusions of propionic acid induces oxidative stress and neuroinflammation in rats [14, 15]. These studies demonstrate that metabolites are critical signaling molecules produced by bacteria and utilized by the host, yet there is limited data on the contribution of gut-derived bacterial metabolites on the earliest stages of the neurodevelopment. Here, we use zebrafish neurodevelopment as a proxy for evaluating the contribution of metabolites to early neural development and gene expression.

## Materials and methods

### Zebrafish maintenance

Zebrafish from the standard wild-type Tübingen (TU) line were raised and maintained in accordance with the Animal Protocol Utilization # 3614 using standard protocols [16]. Zebrafish were maintained on 14:10-h light dark cycle. Larvae were obtained by natural spawning and cultured in zebrafish embryo medium (EM; 0.00006 w/v% Instant Ocean® Sea Salt solution and 0.0001% methylene blue in purified distilled water) at 28.5°C. For in vivo imaging and head dissection, larvae were anesthetized with 0.04% tricaine.

### Generation and treatment of germ-free larvae

Larvae are collected within 2 h of fertilization and develop in a 28.5°C incubator. At shield stage to 60% epiboly (specification of the 3 germ layers but before neurogenesis), the larvae are divided into conventionally raised (CV) and germ-free (GF) groups. CV larvae are left at room temperature (RT) while the GF group is sterilized at RT to normalize their development. GF larvae are immersed in filter sterilized Gentamicin (100𝜇g/mL) for 1 h and subsequently washed in 0.003% hypochlorite followed by three 5-min washes in sterile embryo medium. Embryo treatment is performed under a laminar flow hood to ensure sterility. Post-sterilization, the larvae from both groups are placed in a 28.5°C incubator. After 24 h, a 20-μL sample of both EM and a single homogenized embryo are plated on separate brain heart infusion (BHI) agar plates, a non-selective, nutrient-rich growth medium, along with an empty control plate (exposed concurrently with samples) and incubated at 28.5°C or 37°C for 24 h to test for sterility. Upon confirmation of sterility (0 visible colonies), the larvae are harvested at the appropriate time points outlined below.

### Whole mount in situ hybridization (WMISH)

Zebrafish larvae for WMISH were treated with sterile PTU (0.003%) at 24 h post-fertilization (hpf) to reduce pigment development, harvested at 2, 4, or 5 days post-fertilization (dpf), manually dechorionated, and immediately fixed overnight in 4% paraformaldehyde (PFA, Sigma-Aldrich) in 0.01M phosphate-buffered saline (PBS). WMISH was performed as previously described [17]. DIG-labeled probes were synthesized by in vitro transcription (New England BioLabs Inc.) with appropriate polymerases, following the manufacturer’s instructions and after plasmid linearization with appropriate restriction enzymes.

### Imaging

WMISH-stained larvae were mounted in 100% glycerol. Live larvae were anesthetized in 0.04% tricaine, embedded in 2% methyl cellulose, and imaged with dissecting microscope (V8 Zeiss) mounted with a MicroPublisher 5.0 camera and imaged using Q-Capture software (v 3.1.3.10). Fluorescent images were captured using a Leica CLSM SP5 confocal microscope using LAS AF imaging software v2.7.7.

### Zebrafish metabolites

#### Extraction

Pools of ten adult male zebrafish were euthanized in an ice bath slurry for at least 10 min according to standard procedures [18], followed by surgical removal of the intestine. The intestines were resuspended in sterile 1X PBS at a 1:3 weight to volume ratio (~1 mL) and vortexed for approximately 1 min to resuspend intestinal contents followed by centrifugation at 14,000×g for 30 min. The supernatant was filter sterilized through a 0.22-𝜇M filter and stored at −20°C. To ensure the samples were GF, zebrafish metabolite (ZM) treated egg water was plated as described above and only used if there were no visible colonies after 24h at 28.5°C and 37°C.

#### Treatment

Germ-free larvae were immediately treated with undiluted zebrafish metabolites added directly into the sterile embryo medium by adding 200uL (equivalent of 2.7 adult guts worth) of metabolites mixed with a 15-mL EM in a 10-cm sterile dish containing ~100 larvae at ~60% epiboly. After 24 h, a 20-μL sample of both EM and a single-homogenized embryo were tested for sterility as described above.

### RNA sequencing and analysis

At 2 dpf, the larvae were euthanized in 0.04% Tricane and the heads were surgically removed from the body at the base of the hindbrain. The RNA was extracted from a pool of five heads for each treatment using the GENEzol™ TriRNA Pure Kit (FroggaBio). RNA samples were DNase-treated using the Invitrogen™ DNA-free™ DNA Removal Kit (Thermo Fisher Scientific). An RNA integrity number (RIN) of more than 8.0 was confirmed for all samples using the 4200 Tapestation system (Agilent). Poly(A) mRNA was prepared using the NEBNext® Ultra™ II Directional RNA Library Prep Kit for Illumina® (New England BioLabs), and 2 × 100bp paired-end sequencing at a depth of 80–100 million reads per sample was performed using the Illumina Novaseq 6000 platform by the University of Toronto Donelly Sequencing Centre. FastQC v0.11.8 and HISAT2-2.1.0 [19, 20] were used for quality control and mapping. Reads were aligned to Ensembl Genome Browser assembly ID: GRCz11. Count matrices were created with htseq-count v0.11.0 (ref. [16]), and expression matrices were created with StringTie v1.3.4d [21]. Differential expression analysis was conducted using DESeq2-1.29.13 (ref. [18]). Heatmaps were generated using the ComplexHeatmap v2.5.5 package for R. Raw and normalized count plots were created using ggPlot2 v3.3.2 in R. Enrichment term analysis of rescued genes was conducted using DAVID v6.8 (ref .[19]) and plotted using ComplexHeatmap v2.5.5 in R. Functional enrichments nodes were categorized by GO: biological process, molecular function, and cellular component and/or KEGG or Reactome pathways using a false discovery rate (FDR) less than 0.05.

### Quantitative RT-PCR

RNA was extracted as described above. Quantitative RT-PCR (RT-qPCR) with reverse transcription was performed on a the CFX96 Touch Real-Time Detection system (BioRad) using the Luna Universal One-Step RT-qPCR kit (New England BioLabs) and primer sets validated in our lab (Supplemental Table 1). Universal 16S rRNA gene RT-qPCR primers were synthesized according to Clifford et al. (2012) (ref. [20]).

### Transgenic zebrafish

GFAP:GFP zebrafish Tg(*gfap*:GFP)^*mi2001*^ (Bernardos and Raymond, 2006) were kindly provided by Dr. Vincent Tropepe (University of Toronto) and treated as described above.

### Immunohistochemistry

Larvae were fixed at 2 dpf in 4% paraformaldehyde for 2 h and then rinsed in PBS. The larvae were then exposed to proteinase K (10ug ml^-1^ in PBT) for 20 min and rinsed again in PBS with 1% bovine serum albumin, 1% DMSO, and 0.1% TritonX-100 (PBDT). The larvae were blocked in 10% sheep serum in PBDT for 1 h at room temperature and then incubated in mouse anti-alpha acetylated tubulin (Sigma-Aldrich Canada Ltd, Cat: T7451, Clone: 6-11B-1, 1:500) at 4°C for 48 h. After 48 h, the larvae were rinsed 3 times in PBT and then incubated in the secondary antibody (1:1000) in blocking solution (2% sheep serum in PBDT) for 5 h at room temperature. Following incubation, the larvae were rinsed again 3 times in PBT and exposed to Hoechst counterstain (1:10,000) for 10 min at room temperature before being rinsed in PBS. Five to seven larvae were mounted in 0.8% low melting point agar on glass bottomed imaging dish.

### Lateral line screening

Whole, 3dpf and 4dpf, Tübingen larva from each treatment group were incubated in 4ug/ml Diasp (2-Di-4-Asp, Sigma-Aldrich) and 0.3 ug/ml DioC6 (3,3-dihexyloxacarbocyanine iodide, Sigma-Aldrich) in embryo medium for 5 min as per Valdivia et al. 2011 [22]. After 5 min, the larvae were rinsed 3 times in embryo medium, anesthetized in 0.04% tricaine, and mounted in 0.8% low melting point agar containing 0.04% tricaine on glass bottomed imaging dish and immediately imaged by confocal microscopy, as above.

### Scanning electron microscopy

All SEM images were taken of larvae at 3dpf. The larvae were fixed in 4% PFA overnight and then in 2% glutaraldehyde for 30 min. The larvae were then washed three times in SEM phosphate buffer (1:1 mix of 0.07M K_2_PO_4_ and 0.07M NaPO_4_) before being submerged in 1% osmium tetroxide for 30 min. Next, the larvae were dehydrated in a series of ethanol washes of increasing concentrations and three subsequent washes of 100% ethanol. The larvae were critically dried with CO_2_, mounted onto SEM specimen mounts using double-sided carbon adhesive tape, and sputter-coated with Au/Pd. The larvae were imaged on an FEI Quanta FEG 250 scanning electron microscope.

## R**esults**

### Microbes are necessary for timely neural gene expression

**Fig. 1:**
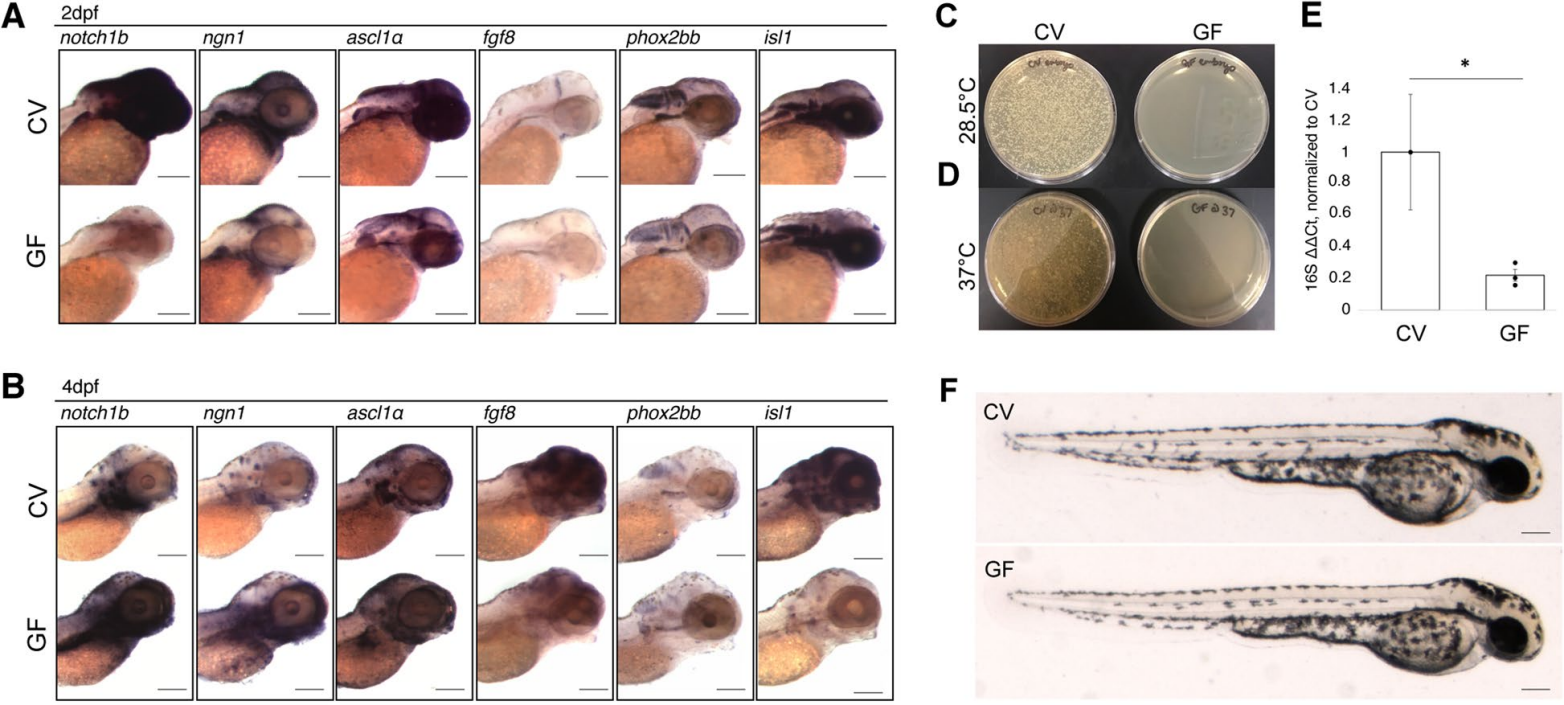
Microbes are necessary for timely neural gene expression. **A** WMISH of target genes in conventionally raised (CV) and germ-free (GF) embryos at 2dpf. RNA expression of target genes *notch1b* (*N* = 6) *ngn1* (*N* = 3), *ascl1a* (*N* = 2), *fgf8* (*N* = 6), and *phox2bb* (*N* = 1) is reduced in the absence of microbes at 2dpf. Expression of *isl1* (*N* = 3) shows no appreciable difference between groups. **B** WMISH of target genes in conventionally raised and germ-free embryos at 4dpf. RNA expression of target genes *notch1b* (*N* = 4), *ngn1* (*N* = 2), and *ascl1a* (*N* = 1) shows an increase in expression in the GF group compared to their CV counterparts at 4dpf. Expression of *fgf8* (*N* = 2) and *phox2bb* (*N* = 2) remains reduced in comparison to the CV group **C**, **D** Whole homogenized single CV (left) or GF (right) embryos plated on brain heart infusion media and left at **C** 28.5°C or **D** 37°C for 24 h. **E** RT-qPCR analysis of universal 16S rRNA gene in CV and GF embryos (* = *p* < 0.05 in a one-way ANOVA, based on delta, delta Ct), normalized to ef1α (error bars represent SEM). RNA was extracted from a pool of five embryos for each group and experiment was conducted in triplicate. **F** Live images of 2dpf zebrafish embryos for morphological comparison. *N* values represent the number independent biological replicates each of which contained approximately 8–10 embryos. Scale bars represent 100um

To determine if microbes are required for neural gene expression and patterning, the spatial distribution of select neural genes were analyzed using whole mount in situ hybridization (WMISH) in conventionally raised (CV) and germ-free (GF) zebrafish embryos. All embryos in each cohort were raised in parallel, were time and stage-matched, randomly assigned in the WMISH protocol, and processed in parallel to ensure that differences in gene expression were not due to an offset in overall development or procedure. The WMISH data demonstrated a significant decrease in expression of five out of six target genes in germ-free embryos in 2 days post-fertilization (dpf) (Fig. 1A). All target genes, except for *isl1*, showed a decrease in relative level of expression. However, expression of *notch1b*, *ngn1*, and *ascl1a*, which had reduced expression levels at 2 dpf, was increased in 4 dpf germ-free embryos, suggesting a delay in expression of these genes under germ-free conditions (Fig. 1B). Expression levels of *fgf8* and *phox2bb* remain decreased in the GF group at 4 dpf relative to CV controls while *isl1*, which showed little difference between treatment groups at 2 dpf, showed a significant decrease in expression in the GF group at 4 dpf. Interestingly, it is the genes that are more ubiquitously expressed that display a delay in expression rather than an overall decrease, yet there are no obvious gross morphological differences between conventionally raised and germ-free zebrafish (Fig. 1F). To determine if the sterilization treatment itself caused the decrease in expression, we exposed GF embryos to the system water from which they were taken immediately after the GF protocol, which rescued gene expression (Supplemental Fig. 1). Taken together, this suggests that there is a delay in neural development in the absence of microbes and their metabolites. We confirmed the sterility of germ-free embryos via homogenizing embryos and plating them on nutrient rich growth medium and incubated at both 28 or 37°C (Fig. 1C, D) and via qPCR of the universal 16S rRNA gene (Fig. 1E). Only when both plates were completely devoid of any bacterial growth did we consider them GF.

### Lack of microbes results in global decrease in neural gene expression

The general decrease in the majority of our WMISH probes suggests that microbes and/or their metabolites might have a more general role in neural development. To determine this, we performed RNA-Seq analysis on RNA enriched from zebrafish heads under three different conditions. As above, we analyzed the gene expression in zebrafish embryos that were conventionally raised and germ-free. To determine if bacterial metabolites were sufficient to affect gene expression, we treated GF embryos at shield stage to 60% epiboly with metabolites isolated and filter sterilized from adult zebrafish guts (ZM). We also conducted 16S rRNA gene sequencing on bacteria isolated from adult zebrafish guts (before metabolite extraction) and a sample of bacteria concentrated from zebrafish spawning tanks (Supplemental Figs. 2 and 3; Supplemental Table 2). We found that the two major phyla in the two samples were the same: Fusobacteria and Proteobacteria, however, the diversity at the genus level was substantially higher in the water sample compared to the whole gut sample for which there was little overlap. We attribute the lower overall diversity and abundance of counts from the gut to the simple fact that we cannot enrich specifically for the colon. Nonetheless, the reduced counts, diversity, and lack overlap from the gut correlates well with the reduced rescue of expression in the ZM samples compared to the CV samples and further suggests that different sources of metabolites (host, environment) are effective in altering neural gene expression.

Total RNA was extracted from the heads of zebrafish embryos at 2 dpf, the height of neurogenesis, enriched for mRNA and subjected to RNA-seq analysis. These experiments are predicated on the assumption that metabolites can pass through the GF treated chorion at 60% epiboly. While the chorion is assumed to be a biological barrier to maintain sterility, there is significant evidence to suggest that metabolites can pass through this membrane.

Secondly, Chen et al. (2020) found that the size of the chorion pore is ~0.77 uM and we used a 0.22-uM filter to sterilize the metabolites. Third, the common SCFA such as butyrate (molecular formula C_4_H_8_O_2_) and propionate (molecular formula C_3_H_2_O_2_) have molar masses of 88.1g/mol and 74.08g/mol, respectively, which is significantly smaller than the 3000 dalton diffusion limit of the chorion. Finally, the routine laboratory compounds such as 1-phenyl 2-thiourea (PTU; molecular formula C_7_H_8_N_2_S) are added prior to 1dpf to inhibit pigment formation. Not only does PTU pass through the chorion, it must also pass through cell membranes.

**Fig. 2:**
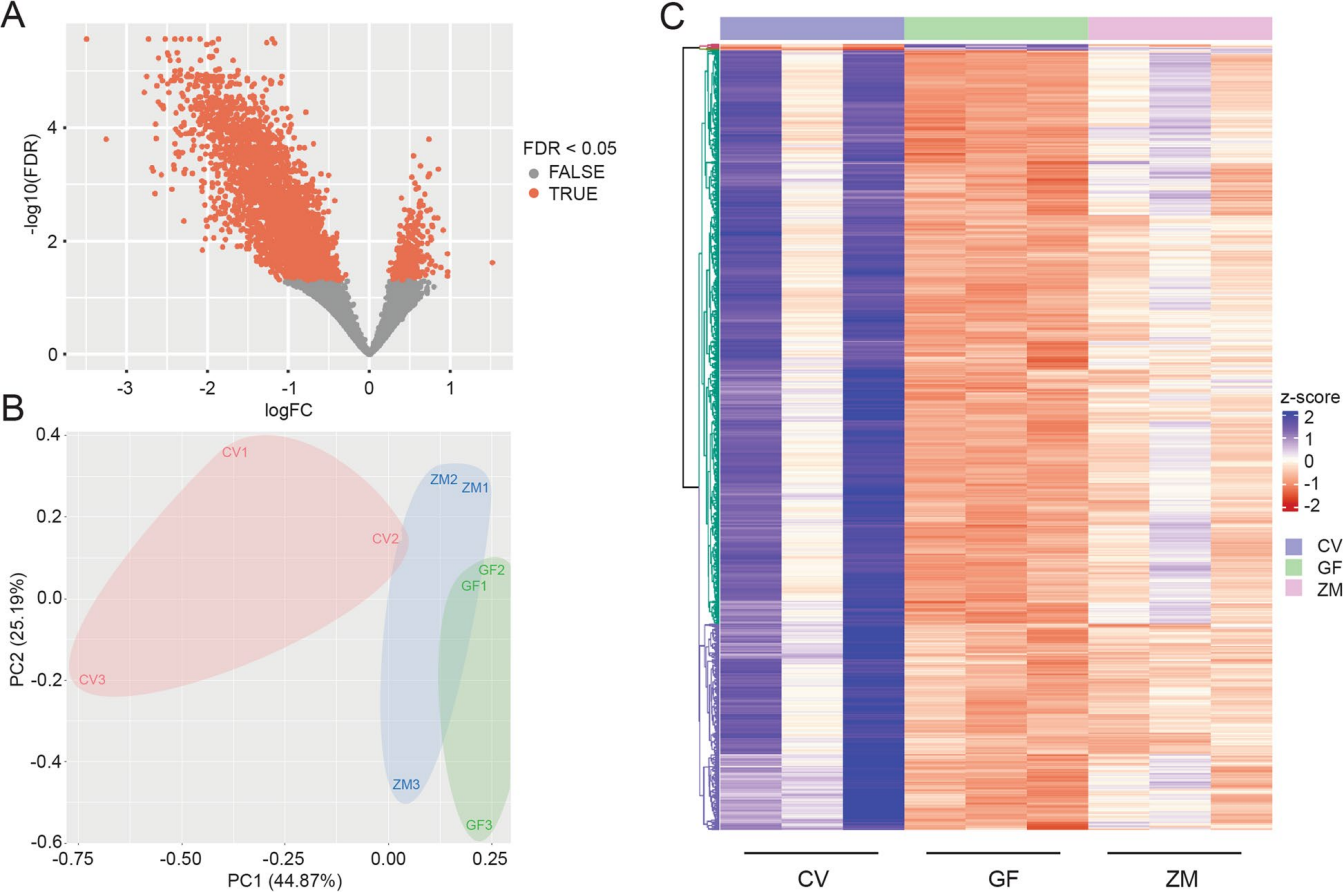
Microbes are both necessary and sufficient for general gene expression in the developing nervous system. **A** Volcano plot comparing DEGs between germ-free larvae and conventionally raised larvae. **B** Principal component (PC) analysis of count data from CV, GF, and ZM zebrafish larvae at 2dpf. Numbers 1, 2, and 3 represent the biological replicates. **C** Heatmap of top 1000 differentially expressed genes between 3 biological replicates of CV, GF, and GF treated with zebrafish gut metabolites (ZM) embryos at 2dpf. Generated with DeSeq2 and ComplexHeatmap

Our first observation was that differential gene expression analysis revealed a general decrease in gene expression in the GF group (Fig. 2A) with over 2000 genes displaying a decrease in expression in the GF group compared to CV (log fold change < 0). Secondly, we observed a substantial decrease in the variation of expression in GF compared to the other treatments (Fig. 2B). Importantly, ZM treatment sufficiently rescued gene expression in GF larvae, along with an increase in the variability (Fig. 2B, C). While the ZM group did not achieve the levels of expression of the CV group, we must consider the short half-life of metabolites, which is on the order of minutes to hours [23, 24]. To test this, we retreated the ZM group with an additional dose of metabolites at 1 dpf and observed an increase in *axin2* expression, consistent with this hypothesis (Supplemental Fig. 4). The variation in the CV and ZM groups compared to the reduced variation in the GF group suggests that in the GF state, there is a basal level of expression that metabolites enhance to varying degrees. Taken together, this suggests that in the absence of microbes, gene expression is uniformly maintained at a seemingly basal level and that metabolites are both necessary and sufficient to elevate or enhance gene expression.

In order to look more specifically at the biological processes and molecular functions associated with germ-free treatment, gene ontology (GO) analysis was performed on the subset of genes whose expression was downregulated at least two-fold. The absence of microbiota resulted in a significant decrease in expression of 354 genes (log fold change ≤2, FDR <0.05) (Supplemental Table 3). GO statistical overrepresentation tests revealed that these genes are largely involved in RNA binding, DNA binding and modification, transcription regulation, neurogenesis, axonogenesis, and Wnt signaling (Supplemental Table 4). It should be noted that there were also six genes upregulated in the GF group compared to CV (log fold change >1); however, these genes did not have any biological significance in statistical overrepresentation tests. These six genes are *serpinh1b*, *crygm5*, *mhc1lfa*, *lenep*, *CU69693494.2*, and *BX000438.2*.

### Metabolites are sufficient to rescue the expression of neural development genes

**Fig. 3:**
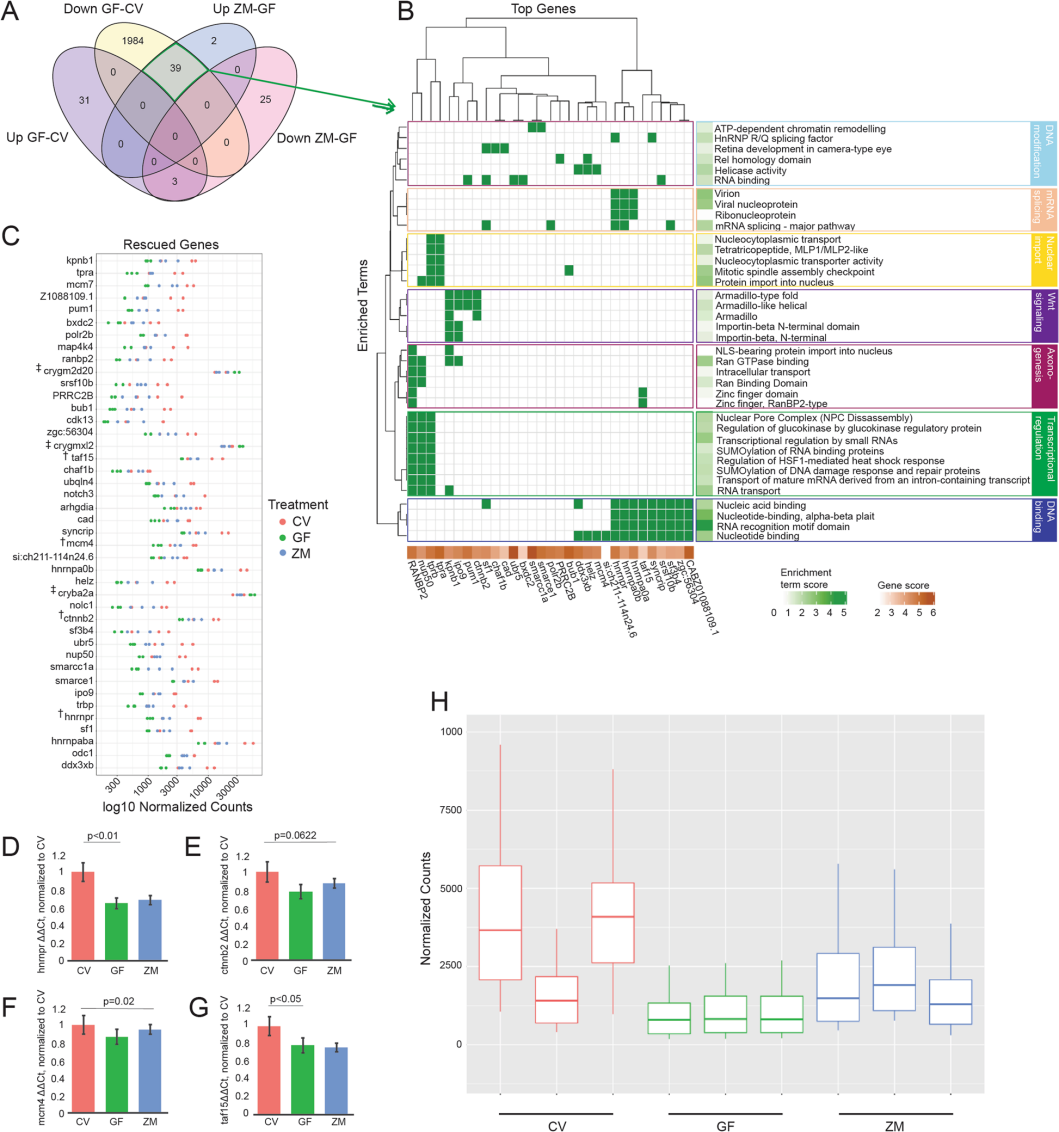
Metabolites are sufficient to rescue neural gene expression in GF larva. **A** Venn-diagram comparing gene expression levels between CV and GF and those rescued by the addition of zebrafish metabolites to GF embryos (ZM-GF) (p < 0.05; GF-CV FDR < 0.05, ZM-GF FDR < 0.1). **B** DAVID generated plot of 31 of the 39 rescued genes and their associated enrichment terms (note: 8 genes did not contribute to significant over representation in DAVID output). Enrichment terms largely fall within seven major biological processes noted on the right. **C** Normalized counts of all 42 genes (39 downregulated plus 3 upregulated, noted by ‡ ) whose expression was rescued with metabolite treatment. Count comparison and RT-qPCR validation (**D–G)** of four rescued genes (marked with † in C from the RNA-seq dataset. RT-qPCR validation of hnrnpr (**D**), ctnnb2 (**E**), mcm4 (**F**), and taf15 (**G**) (one-way ANOVA of all 3 groups’ standard weighted mean analysis, 3 independent samples, 2 degrees of freedom, total p value is as stated, error bars represent SEM). **H** Boxplot of normalized counts of 39 downregulated and rescued genes between treatment groups with outliers removed. Top and bottom of box represents the 75th and 25th percentile respectively. The 50th percentile and solid horizontal line in the box represents the median. Whiskers represent the largest and smallest value within 1.5 times interquartile range above 75th percentile and below 25th percentile, respectively

The addition of metabolites to germ-free zebrafish rescued the expression of numerous genes that were significantly downregulated in GF (*p* <0.05, FDR <0.05) compared to CV larvae, although not to the extent observed in CV larvae. We considered gene expression to be rescued by zebrafish metabolites if the log fold change of a gene in ZM-GF was in the opposite direction of the log fold change of the same gene in GF-CV (GF-CV FDR <0.05, ZM-GF FDR <0.1). Using these criteria, the expression levels of 42 genes were rescued by metabolites (Fig. 3A). That is, 39 genes were downregulated in the GF group compared to CV but upregulated in the ZM group compared to GF, and 3 genes were upregulated in the GF group compared to CV and downregulated in the ZM group compared to GF (Fig. 3A, C). The expression levels of these 39 upregulated genes were highly variable between the 3 CV biological samples (Fig. 3H), consistent with the analysis of the entire data set (Fig. 2B, C). Interestingly, this variation was considerably reduced in the GF samples, but increased again upon treatment with metabolites.

We analyzed the function of these 39 genes using DAVID, an online bioinformatic tool that condenses gene lists and associated biological terms for functional annotation using four analysis modules: Annotation Tool, GoCharts, KeggCharts, and DomainCharts (https://david.ncifcrf.gov/). The output from DAVID was plotted via ComplexHeatmap v2.5.5 package for R (Fig. 3B, Supplemental Table 5). Of the 39 downregulated rescued genes, 30 had biological significance in a gene function analysis in DAVID (Fig. 3B). The genes rescued by metabolites are largely involved in cellular processes related to DNA binding, nuclear import, transcriptional regulation, and mRNA splicing, as well as neural developmental processes involving Wnt signaling and axonogensis. Overall, this emphasizes the importance of metabolites during early neural development. The three genes that were downregulated and rescued, *cryba2a*, *crygmxl2*, and *crybm2d20*, are associated with eye and lens development and were not included in the DAVID plot but are included in the normalized count plot (Fig. 3C). Curiously, these 3 genes had significantly higher levels of expression in CV compared to the others and the changes in expression, while significant, were to a smaller degree compared to the genes whose expression were upregulated in ZM. To validate the RNA-seq data, select genes from this list were quantified via RT-qPCR from independent sources of mRNA for the three conditions (Fig. 3D–G). These results support both the RNA-seq and WMISH data that metabolites are both necessary and sufficient for gene expression.

### Neural development is disrupted in germ-free embryos

**Fig. 4:**
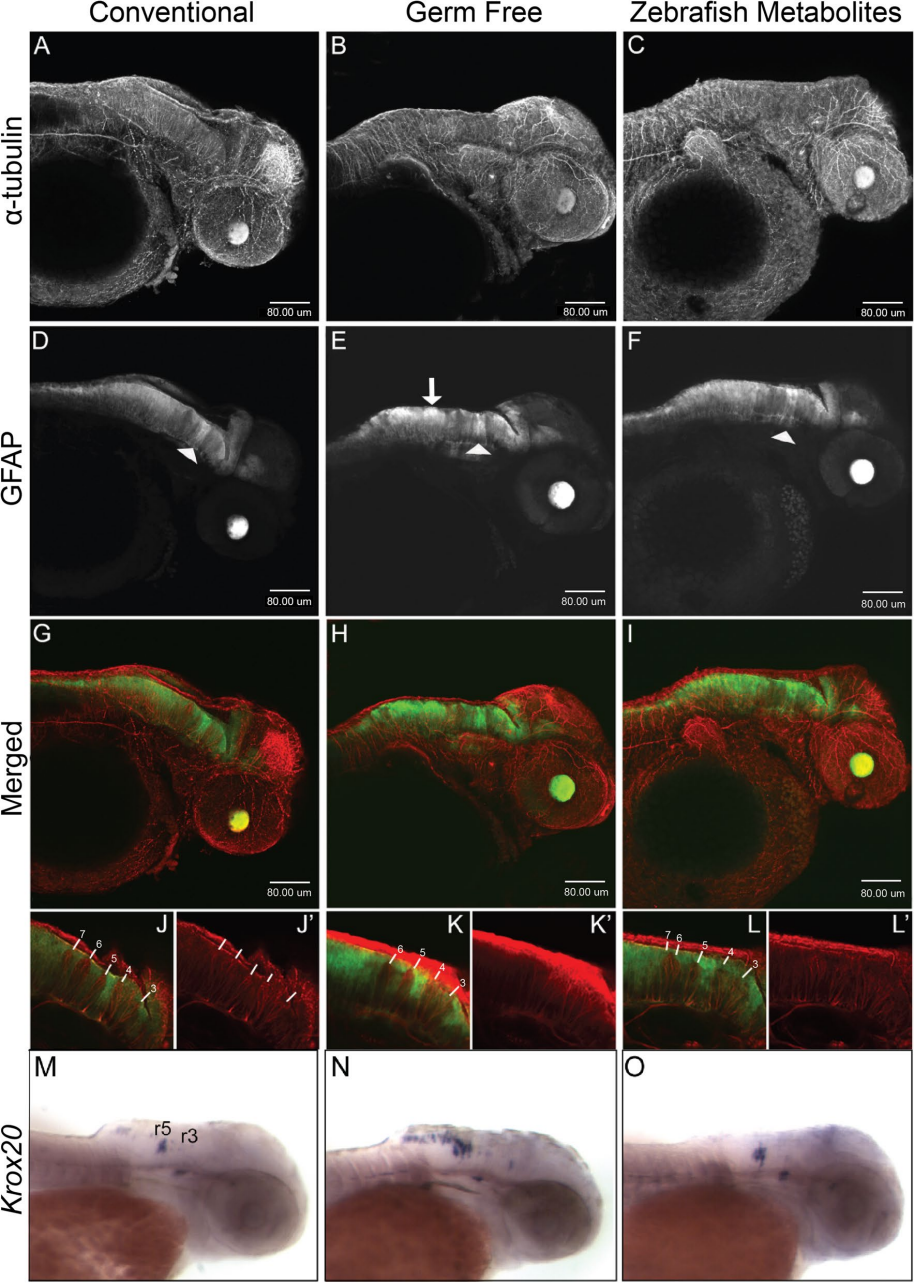
Neural development is disrupted in germ-free embryos. Confocal projection images of zebrafish embryos at 2dpf. **A–C** α-tubulin immunostaining. **D–F** GFAP:GFP fluorescence displays a non-uniform distribution in the hindbrain in germ free embryos (white arrow in **E**) and to some extent in ZM-treated embryos. The white arrowheads identify the GFAP tract between rhombomeres 4 and 5 which do not appear to be significantly altered in germ-free embryos. **G**–**I** Merged images of α-tubulin and GFAP to GFP. **J**–**L** Representative single-layer images of regions in the hindbrain. In conventional embryos, rhombomere tracts, 3–7 are readily identifiable by the relative absence of GFAP fluorescence. The higher intensity GFP to GFAP fluorescence between rhombomeres 4 and 5 provides a landmark for their easy identification. Note the absence of rhombomere 7 in germ-free embryos, and the seemingly merged tracts 6 and 7 in ZM-treated embryos. More examples are presented in Supplementary Figs. 5 and 6. **M-O** WMISH of *krox20* in 2dpf embryos from CV (**M**), GF (**N**) and ZM treated (**O**) embryos. Rhombomeres 3 (r3) and 5 (r5) are labelled. Larvae from each treatment group were processed in parallel

The absence of a gross morphological phenotype but significant decrease in gene expression in GF prompted us to investigate the consequence of being germ-free at the cellular level. Using acetylated α-tubulin immunostaining as a general axon marker in combination with transgenic glial fibrillary acidic protein (GFAP:GFP) to mark neural stem cells and glia, we looked at the general architecture of the zebrafish larval brain at 2 dpf (Fig. 4 A–F). Consistent with our WMISH and RNA-seq data, we observed a modest and generalized disorganization of neurons and glia in GF larvae. In particular, we observed an uneven distribution in GFAP-GFP fluorescence in GF compared to CV and ZM treatments (Fig. 4D–F). Upon closer inspection of rhombomeres in the hindbrain, GFAP to GFP fluorescence reveals changes in the pattern of rhombomeres in GF embryos compared with CV and ZM treatments (Fig. 4J–L). To better understand the changes in GFAP immunostaining in the hindbrain, we performed WMISH with Krox20 which labels rhomobomere 5 in 2 dpf embryos (Fig. 4M–O). Expression of rhombomere 3 has significantly decreased expression at this stage. In CV embryos, we observed clear demarcation of krox20 expression in rombomere 5. Consistent with the uneven GFAP immunostaining in GF embryos, we observed a caudal expansion of Krox20 in approximately 50% of GF treated embryos from two independent experiments. This suggests that there is some modification of the hindbrain structures in the GF embryos, but the significance of this remains to be determined. Curiously, unlike the majority of the broadly expressed neural genes shown in Fig. 1, *Krox20* expression was expanded in the GF embryos which was rescued by ZM. However, evaluation of the RNA-seq data showed no significant differences between the treatments, which we speculate may be due to the variability in its expression.

**Fig. 5:**
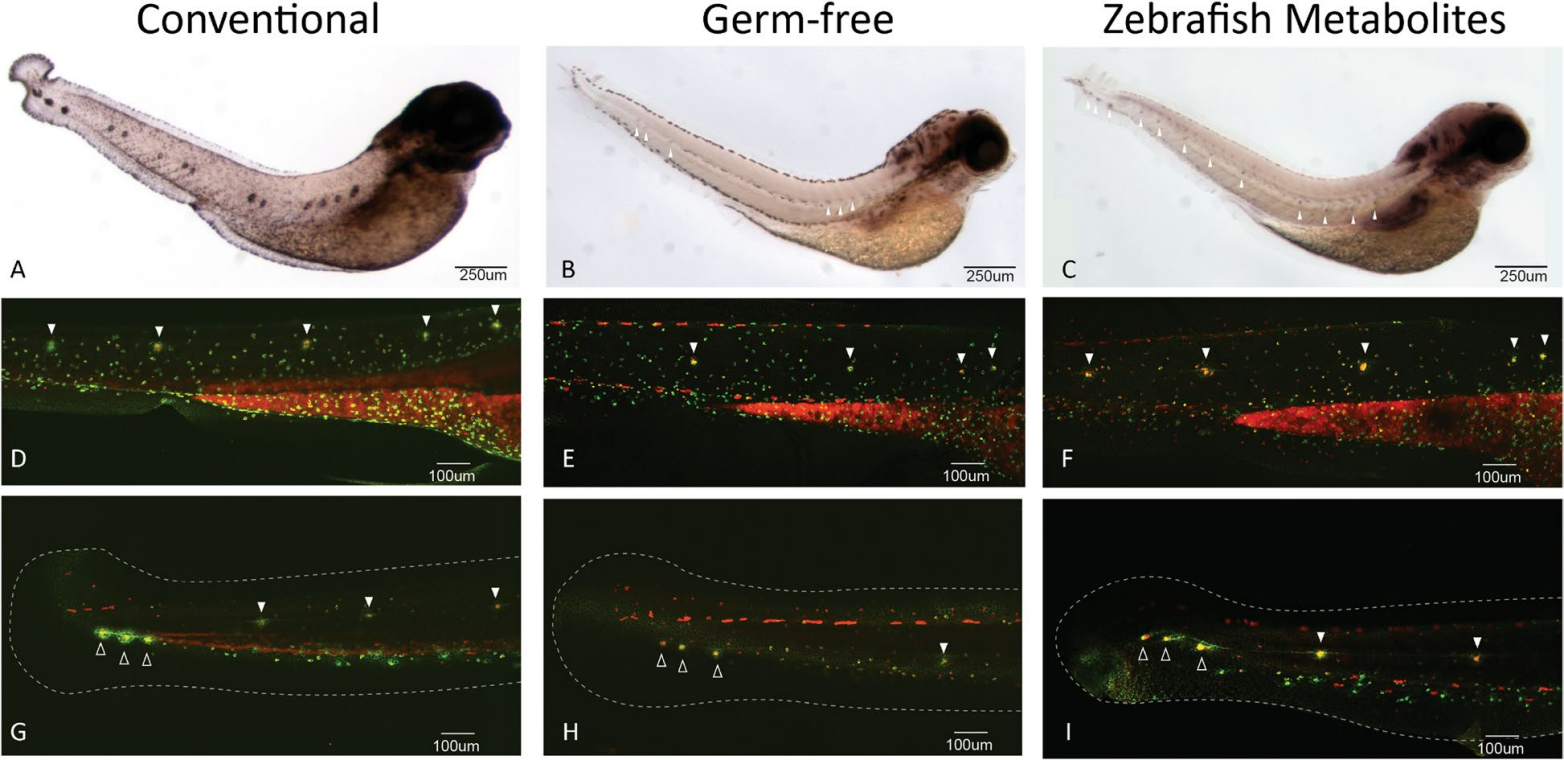
Posterior lateral line development is disrupted in germ-free embryos. **A**–**C** WMISH of isl1 in 4dpf embryos. Larvae from each treatment group were processed in parallel. All groups were stained for the same period of time to allow comparison of GF and ZM groups which have reduced staining, but visible neuromasts (white arrowheads). Experiment was conducted with 8–10 larvae per group. **D**–**F** Trunk neuromasts of the posterior lateral line in 3dpf larvae incubated in a mixture of vital dyes Diasp and DiOC6 to identify hair cells (red) and accessory cells (green) of the lateral line. Neuromasts are marked with white arrowheads. The intense staining in the yolk extension provides a useful positioning reference. **G**–**I** Posterior trunk neuromasts (solid white arrowheads) and terminal neuromasts (hollow arrowheads) in the tail identified with Diasp and DiOC6 in 3dpf. Tail fins are outlined with dashed line for reference. Terminal neuromasts appear to be smaller and less well-developed in GF larvae. Some neuromasts of the posterior tail, as well as some terminal neuromasts, are missing in GF larvae. More examples are presented in Supplementary Figs. 8, 9 and 10

**Fig. 6:**
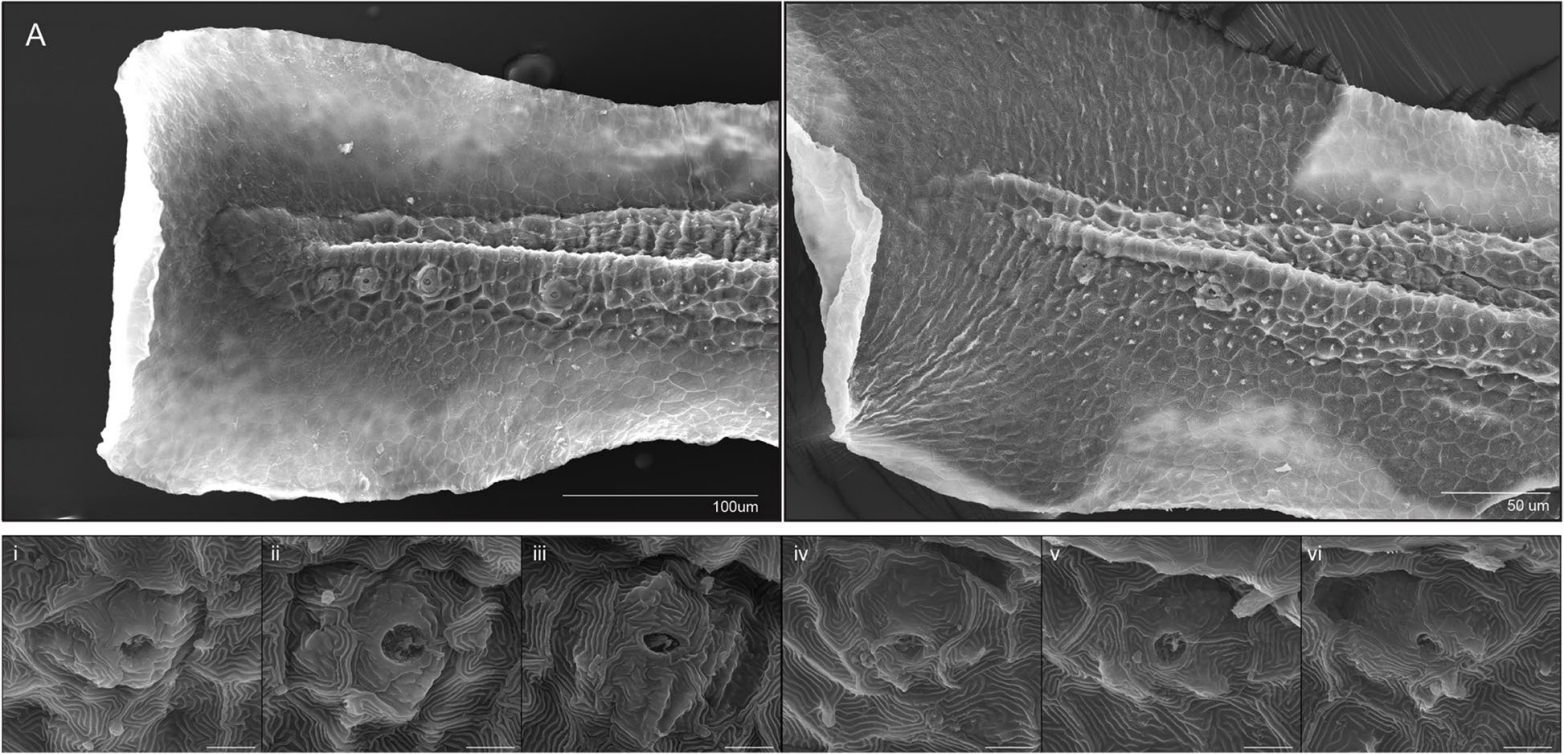
Development of terminal neuromasts is disrupted in germ-free embryos. Scanning electron microscopy of terminal neuromasts of 3dpf larvae. **A** Tail of a CV larvae. **B** Tail of GF larvae. **i–iii** Representative individual terminal neuromasts of CV 3dpf larvae (scale bars = 5um). **iv**–**vi** Representative individual terminal neuromasts of GF 3dpf larvae (scale bars = 5um). Terminal neuromasts of CV larvae had an average aperture diameter of 3.44um, which was significantly larger (*p*<0.01, Student’s *t* test) than GF larvae, which had an average aperture diameter of 2.69 um (standard deviations 0.74 and 0.84, respectively). Average aperture area was also significantly larger (*p*<0.01, Student’s *t* test) in CV larvae at 8.64um compared to an average of 5.00um in the GF group (standard deviations 3.52 and 2.93, respectively) (22 neuromasts imaged from 8 larvae in the CV group, 14 neuromasts from 6 larvae in the GF group)

The zebrafish lateral line is a mechanosensory organ that requires coordination of cell proliferation, migration, and differentiation [22]. Furthermore, it has been well documented that Wnt signaling plays an active role in primordial neuromast deposits of the lateral line [22]. The posterior lateral line of the trunk arises from the first placode at around 18 hpf, which gives rise to neuroblast precursors and the first primordium that migrates down the trunk over the next 20 h depositing cellular rosettes that eventually differentiate into neuromasts [25]. Thus, investigating the lateral line in GF larva would be a useful way to evaluate neural cell migration and specification with potential links to Wnt signaling, which we observed to be perturbed in our RNA-seq analysis in GF larvae. To investigate this, we first looked at neuromasts by WMISH with *ascl1a*, *notch1b*, and *isl1* (Fig. 5A–C; Supplemental Fig. 7) at 4 and 5 dpf. We observed alterations in the location and number of primordial neuromasts of the lateral line in GF larvae, which was partially rescued with ZM (Fig. 5A–C). To further evaluate this, we performed live vital dye analysis with Diasp and DiOC6, which also demonstrated that the development of the posterior lateral line is disrupted in germ-free embryos and rescued to some extent in the embryos treated with zebrafish metabolites (Fig. 5D–I, Supplemental Figs. 8, 9 and 10). At 3 dpf, neuromasts of the posterior lateral line in the trunk of GF embryos appear unevenly distributed, more anteriorly positioned and immature compared to the CV and ZM embryos, where on average, more of the neuromasts in both the CV and ZM groups have migrated past the anal pore, consistent with the WMISH data (Fig. 5G–I, Supplemental Fig. 8). Aside from the obvious change in location and number, it is difficult to accurately quantify these differences. To address this, we performed scanning electron microscopy of 3dpf larvae, which revealed that the terminal neuromasts are less well-developed and in some cases missing in GF embryos (Fig. 6). Measuring the aperture of terminal neuromasts demonstrated that the GF neuromasts are significantly smaller (*p* < 0.01, Student’s *t* test) with approximately 40% smaller aperture area and 20% narrower diameter compared to CV terminal neuromasts (Fig. 6). We also observed changes in posterior lateral line neuromasts at 4dpf, where GF embryos had between one and four trunk neuromasts compared to CV embryos that had between four and seven (Supplemental Fig. 10)

### Metabolites affect Wnt signaling

**Fig. 7:**
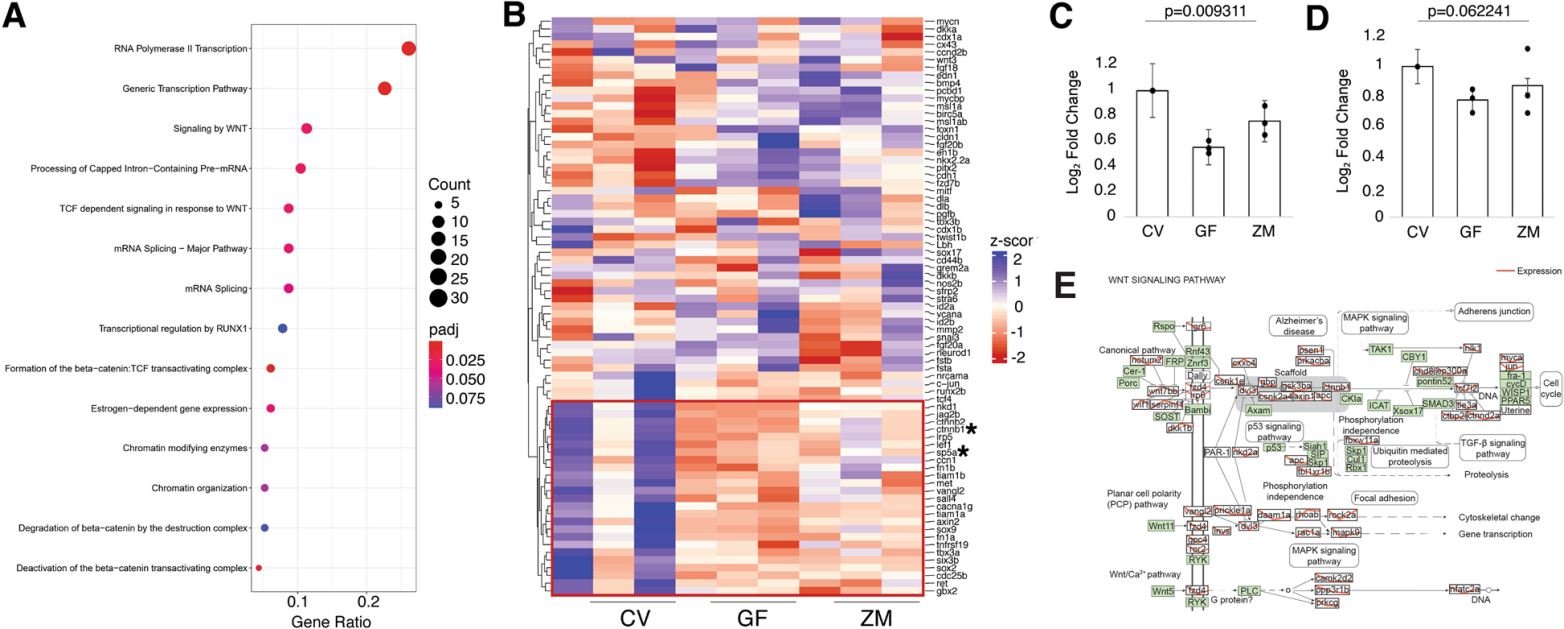
Metabolites affect Wnt signaling. **A** Enriched pathway analysis of the downregulated genes in the GF group via KEGG profile. **B** Heatmap of 75 Wnt/β-catenin signaling genes identified by the Wnt signaling community. Box in red shows genes that are affected in our dataset. Asterisks identify two genes that were validated by RT-qPCR (**C**, *sp5* and **D**, *ctnnb2*; one-way ANOVA of all 3 group standard weighted mean analysis, 3 independent samples, 2 degrees of freedom, total *p* value is as stated, error bars represent SEM). **E** KEGG profile output of Wnt pathway and genes from complete RNA-Seq dataset. Expression levels are shown in red where the left side (CV) is arbitrarily set to 0, the middle point is GF, and the right point is ZM

The combination of the DAVID output (Figs. 3B and 7A) identifying Wnt signaling and the effect of the neuromast development (Figs. 5 and 6), a Wnt-dependent event, as being affected by bacterial metabolites prompted us to further investigate Wnt signaling. Wnt signaling is important in many developmental processes including cell fate determination, proliferation, axonogenesis, and migration [26]. Indeed, there is evidence that bacteria activate Wnt signaling to regulate the inflammatory response [27]. Further, several studies have demonstrated that bacteria activate Wnt signaling with effects on the intestinal epithelium [28–31], reproductive tract [32, 33], and respiratory tract [34, 35]. Studies in both mice and zebrafish have shown that bacteria induce intestinal cell proliferation in a Wnt-dependent manner and that germ-free animals have decreased Wnt signaling and decreased intestinal epithelial cell proliferation [36, 37]. To explore this further, we identified 75 genes that the Wnt community has identified as being targets of, or important in, Wnt/β-catenin signaling (The Wnt Homepage; Fig. 7B). We found that 25 of the 75 genes exhibited reduced expression in GF and rescued expression in ZM pattern. We validated two of these genes (*sp5a* and *ctnnb2*, Fig. 7C, D) and further performed a KEGG analysis (Fig. 7E), all of which demonstrates that the Wnt pathway is one of the major signaling pathways affected by bacterial metabolites. In addition to Wnt signaling, other developmental signaling pathways were also affected, including TGFβ, Hedgehog, and Notch (Supplemental Fig. 11), consistent with the broad decrease in gene expression in the GF treatment.

**Fig. 8:**
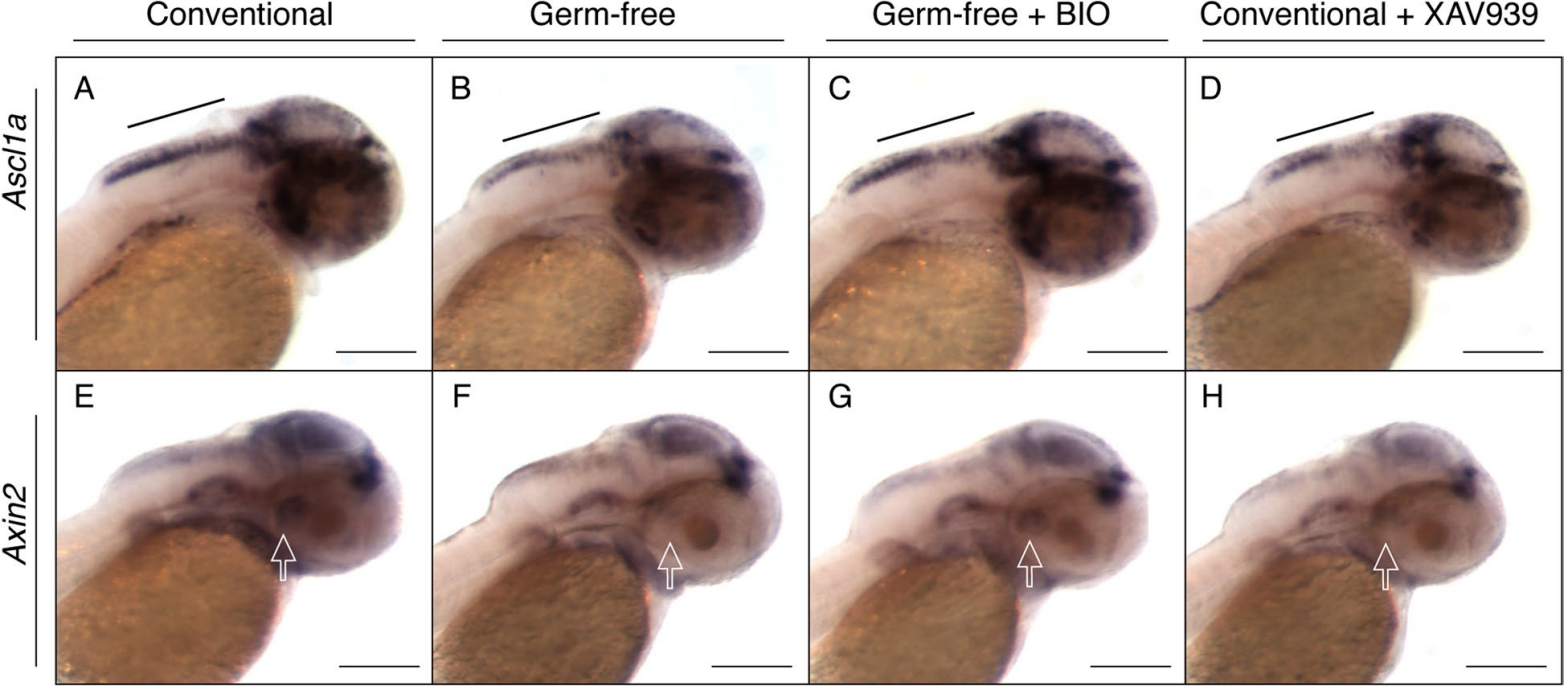
Specific regulation of Wnt signaling mimics the GF and ZM conditions. Representative images of whole mount in situ hybridization on 2 dpf conventional embryos, germ-free embryos, germ-free embryos treated with known Wnt activator BIO, and conventional embryos treated with known Wnt inhibitor XAV939 for genes *ascl1a* (**A–D**) and *axin2* (**E–H**)*.* All embryos were treated in parallel and stained for the same period of time. Black bar identifies the hindbrain region where there is less staining in both the GF and conventional treated with Wnt inhibitor than the CV or GF treated with Wnt activator. Hollow white arrows represent the posterior recess of the hypothalamus [41, 42]. Experiment was conducted using 8–10 embryos per group. Scale bars represent 100um

Because Wnt signaling was affected in our dataset, we investigated whether the decrease in expression of developmental genes was at least in part due to downregulated Wnt signaling. We used two compounds known to affect Wnt signaling to treat CV and GF embryos and analyzed expression of neurodevelopment gene *ascl1a* and Wnt target *axin2* via WMISH. Conventionally raised embryos were treated with XAV939, a small molecule that inhibits Wnt activity [38, 39]. GF embryos were treated with BIO, a compound that functions as a Wnt activator [40]. Each compound was added to either CV or GF embryos, respectively, immediately after the GF embryos were sterilized and all four groups of embryos were allowed to develop to 2 dpf and processed in parallel. Both the GF and the CV + XAV939 treated larvae displayed a relative decrease in expression of both *ascl1a* and *axin2*, consistent with our previous findings. Importantly, the GF + BIO-treated larvae displayed relatively higher expression like that of the CV larvae (Fig. 8). Spatially, the expression was predominantly affected in the hindbrain (Fig. 8A–D) and the posterior recess of the hypothalamus [41, 42] (Fig. 8E–H). Indeed, specifically inhibiting Wnt appears to have the same effect on expression of *ascl1a* and *axin2* as deriving the embryos germ-free. Further, treating GF embryos with a Wnt activator rescues the expression of these genes to a level that is comparable to CV larvae. Taken together, these results suggest that Wnt signaling is dependent on microbes at some level, though more research is necessary to determine causation.

## Discussion

In this study, we evaluated the contribution of bacteria and gut-derived metabolites on neural gene expression and development. Making zebrafish germ-free appeared to have no gross morphological effect at early larval stages, yet demonstrated a significant decrease in gene expression of thousands of genes. Further, the addition of zebrafish gut-derived metabolites to germ-free-treated embryos rescued the expression of several genes and Wnt-dependent activities, thus demonstrating the role of metabolites in neural gene expression and Wnt signaling that is independent of potential antibiotic and hypochlorite-related effects. In other models, germ-free animals initially appear normal but tend to function at a lower metabolic efficiency [43–45] and have negatively impacted development of other organs and organ systems [4, 46–48]. Intestinal microbes provide significant biochemical functions to generate metabolites that eukaryotes are incapable of generating such as butyrate, propionate, and acetate [2]. While there may be no gross morphological effects, we do demonstrate that gut-derived metabolites are in large part responsible for regulating critical signaling pathways in the brain, especially during neural development.

### Large genomic effects

Overall, we observed a general decrease in expression of many genes in GF, which was partially rescued by zebrafish metabolites. Further, we found significant expression level variability in the CV and ZM groups, which was dramatically reduced by making larvae germ-free. This suggests that there is a basal level of expression that is amplified by bacterially derived metabolites. It is interesting that we did not observe gross morphological differences between the treatment groups, which we speculate may be due to the maternal contribution of metabolites in the yolk. These maternally derived metabolites may also contribute to the basal level of gene transcription that we observed. Nonetheless, given that GF gene expression can be rescued by the addition of metabolites provides an attractive platform in which to study the contribution of purified or specific metabolites to biological processes as has been observed in GF mouse models [3]. Further, the hair cells in the lateral line are analogous to mammalian inner ear hair cells and as such provides a tractable model for understanding the contribution of bacterial metabolites to relevant biological processes [49]. We are currently identifying other biological processes that are perturbed in GF larvae and rescued by metabolites to further pursue this model.

### Mining the contributions of metabolites

Deriving zebrafish embryos germ-free resulted in a significant reduction in the expression of 354 genes and an increase in seven genes. Treating GF embryos with metabolites derived from the zebrafish gut significantly rescued the expression of 42 of these genes. Using DAVID analysis, we found that RNA binding, DNA binding, and modification and transcription regulation genes were the major genes being affected by both the absence and addition of metabolites. While the levels of transcription in ZM larvae did not reach CV levels, they were sufficient to rescue defects in the developing nervous system caused by being germ-free. Our findings are consistent with other studies that have demonstrated that microbiome depletion is linked to alterations in RNA processing, particularly alternative splicing [6], and previous studies demonstrating that gut microbiome metabolites can affect DNA and RNA binding, processing, and transport [50–52].

### Wnt signaling/lateral line

We observed that several prominent developmental signaling pathways are responsive to gut metabolites, most notably Wnt signaling. Indeed, when we enriched for Wnt signaling genes identified by the Wnt community, we found a significant reduction in 25/75 of these in GF larvae. Wnt signaling is well-recognized for its role in neural development [26], posterior lateral line [22, 53], and mental disorders [54]. Interestingly, we found similar results via WMISH of *axin2* in 2 dpf GF embryos and CV embryos treated with a Wnt inhibitor as a recent report of hypothalamic genes associated with Wnt signaling and anxiety in a zebrafish Lef1 mutant [41]. As Wnt signaling is also influenced by bacteria [27], it is not surprising that we observed alterations in Wnt signaling-dependent processes. Wnt-dependent activities, such as the migration and development of the lateral sensory hair cells, were affected in GF and rescued in ZM. The uniform distribution of GFAP in CV larvae was also disrupted in the GF treatment and rescued by the ZM treatment. GFAP is a marker of neural stem cells and glia, and we observed an increase in GFAP:GFP fluorescence in GF larvae, which is consistent with the delay in neurogenesis that we observed by WMISH and seen in Wnt1 morpholino knockdown studies [36].

Independent studies have also demonstrated that Wnt signaling was downregulated in germ-free mice, which displayed defects in thalamocortical axonogenesis and aversive somatosensory behaviors [3]. Further, the Wnt/β-catenin effector Lef1 is required for the development of the hypothalamus and differentiation of anxiolytic hypothalamic neurons in both zebrafish and mice, which also displayed increased anxiety in zebrafish in the absence of Wnt/β-catenin signaling [41]. Taken together, there is strong evidence that metabolites are directly regulating Wnt signaling, which impinges on several neurodevelopmental processes.

### Comparison to other studies

Our expression results are consistent with previous reports in microbiome depleted mice. A recent study by Vuong et al. (2020) found that microbiome depletion altered the expression of 333 genes in the brains of embryonic mice, including many genes involved in axonogenesis. We found 67 of the same genes differentially expressed in GF zebrafish embryos. Somewhat surprisingly, one of the genes rescued by metabolites in both the Vuong et al. (2020) study and in the current analysis is *ctnnb2*, CTNNB1, the central contributor to the Wnt signaling pathway, which has been implicated in other studies looking at specific microbial species [29, 55, 56]. Independent of germ-free status, both Wnt signaling and axonogenesis have been implicated in studies of the microbiome [3, 27, 37, 57]. We also found a substantial overlap between differentially expressed genes in the current dataset and genes identified as candidate risk genes for neurodevelopmental disorders, where 256 genes that were downregulated in GF larvae compared to CV larvae are orthologous to genes identified by SFARI (Supplemental Table 6). The independent and consistent identification of Wnt signaling as a target of bacterial metabolites, the well-established role of this pathway in neural development, and the role this pathway plays in so many diseases, elevates this pathway to a new level. Further, the comparison of germ-free animal models should ultimately identify a universal set of genes most likely affected by metabolites.

## Conclusion

It is becoming quite clear that neural development does not occur in a sterile and metabolite-free environment. However, understanding how these metabolites impinge on neural development is still in its infancy. Consistent with other independent investigations, we identified significant changes in neural gene expression that are under the influence of bacterially derived metabolites. With such substantive changes, it can be difficult to identify the most important players, but the Wnt signaling pathway has emerged as playing a leading role in this process. Given that this pathway first arose in multicellular eukaryotes and plays such a significant role in development and disease, perhaps it should not be surprising that its regulation co-evolved with the bacterial colonization of multicellular eukaryotes. Further investigation into the metabolite-Wnt-neurodevelopment axis could ultimately lead to better therapies for the myriad of Wnt-related mental disorders [54].

Key resource table

**Table:** 

Reagent or resource type	Designation	Source	Identifier
Strain	Tg (*gfap*: GFP)^*mi2001*^		ZFIN ID: ZDB-ALT-060623-4
Antibody	Monoclonal anti-acetylated tubulin antibody produced in mouse	Sigma-Aldrich Canada Ltd.	Cat: T7451, Clone: 6-11B-1
Antibody	Donkey anti-mouse IgG (H+L) Alexa Fluor 594	Thermo Fisher Scientific	A-2120; RRID AB_141633
Vital dye	2-Di-4-Asp	Sigma-Aldrich Canada Ltd.	Cat: D3418
Vital dye	3,3-dihexyloxacarbocyanine iodide	Sigma-Aldrich Canada Ltd.	Cat: 318426

## Supplementary Information

**Additional file 1: Supplemental Table 1.** Excel doc of primer set sequences and efficiencies.  **Supplemental Table 2.** Amplicon Sequence Variant (ASV) table of sequence abundance and taxonomy in zebrafish gut sample versus zebrafish water sample. **Supplemental Table 3**. List of 354 DE genes (downregulated in GF-CV). **Supplemental Table 4.** GO output. **Supplemental Table 5.** David output. **Supplemental Table 6.** List of DE genes that are also SFARI genes

**Additional file 2: Supplemental Figure 1.** WMISH of *notch1b* in 2dpf embryos that were derived germ-free and then reintroduced to CV embryo medium. **Supplemental Figure 2.** Relative Abundance of bacteria in zebrafish gut sample versus zebrafish water sample at the phylum level. Taxonomy was assigned using a training set of reference sequences via the Silva 138.1 prokaryotic SSU taxonomic training data formatted for DADA2. **Supplemental Figure 3**. Relative Abundance of bacteria in zebrafish gut sample versus zebrafish water sample at the genus level. Taxonomy was assigned using a training set of reference sequences via the Silva 138.1 prokaryotic SSU taxonomic training data formatted for DADA2. **Supplemental Figure 4.** WMISH of *axin2* in 2dpf embryos that were derived germ-free and then treated once, or twice with zebrafish metabolites. **Supplemental Figure 5.** Single layer composite of axonal tracks. **Supplemental Figure 6.** Projected images of a-tubulin and GFAP:GFP expression. 3dpf lateral line composite trunk. **Supplemental Figure 7.** WMISH of 4 and 5 dpf larvae with *notch*, *ascl1a* and *isl1* **Supplemental Figure 8.** 3dpf lateral line composite. **Supplemental Figure 9.** 3dpf lateral line composite of posterior lateral line. **Supplemental Figure 10.** 4 dpf lateral line composite of posterior lateral line. **Supplemental Figure 11.** KEGG images of various signalling pathways.

## Data Availability

Data generated or analyzed during this study are included in this published article [and its supplementary information files]. Complete datasets generated for RNA sequencing during and/or analyzed during the current study are available in the NCBI GEO expression omnibus, accession: GSE182725. ^**^Data is set to private during review. To review GEO accession GSE182725: Go to https://www.ncbi.nlm.nih.gov/geo/query/acc.cgi?acc=GSE182725. Enter token ybojmeiqdbkpryr into the box.

## Abbreviations

ENS: Enteric nervous system
GABA: Gamma amino butyric acid
SCFA: Short-chain fatty acid
BBB: Blood-brain barrier
EM: Embryo medium
WMISH: Whole mount in situ hybridization
CV: Conventionally raised
GF: Germ-free
RT: Room temperature
BHI: Brain heart infusion
HPF: Hours post-fertilization
DPF: Days post-fertilization
ZM: Zebrafish metabolites
PFA: Paraformaldehyde
PBS: Phosphate-buffered saline
RIN: RNA integrity number
FDR: False discovery rate
PTU: Phenythiourea
GO: Gene ontology
TNKS: Tankyrase
DEG: Differentially expressed genes
