# Supplementary material for: Gut-derived metabolites influence neurodevelopmental gene expression and Wnt signaling events in a germ-free zebrafish model
Source: Microbiome. 2022 Aug 23;10:132. doi: 10.1186/s40168-022-01302-2 (PMC9396910; doi:10.1186/s40168-022-01302-2)
Supplement: Supplementary file 3 — Additional file 2: Supplemental Figure 1. WMISH of notch1b in 2dpf embryos that were derived germ-free and then reintroduced to CV embryo medium. Supplemental Figure 2. Relative Abundance of bacteria in zebrafish gut sample versus zebrafish water sample at the phylum level. Taxonomy was assigned using a training set of reference sequences via the Silva 138.1 prokaryotic SSU taxonomic training data formatted for DADA2. Supplemental Figure 3. Relative Abundance of bacteria in zebrafish gut sample versus zebrafish water sample at the genus level. Taxonomy was assigned using a training set of reference sequences via the Silva 138.1 prokaryotic SSU taxonomic training data formatted for DADA2. Supplemental Figure 4. WMISH of axin2 in 2dpf embryos that were derived germ-free and then treated once, or twice with zebrafish metabolites. Supplemental Figure 5. Single layer composite of axonal tracks. Supplemental Figure 6. Projected images of a-tubulin and GFAP:GFP expression. 3dpf lateral line composite trunk. Supplemental Figure 7. WMISH of 4 and 5 dpf larvae with notch, ascl1a and isl1 Supplemental Figure 8. 3dpf lateral line composite. Supplemental Figure 9. 3dpf lateral line composite of posterior lateral line. Supplemental Figure 10. 4 dpf lateral line composite of posterior lateral line. Supplemental Figure 11. KEGG images of various signalling pathways. [file 40168_2022_1302_MOESM2_ESM.pdf]

notch1b - 2dpf

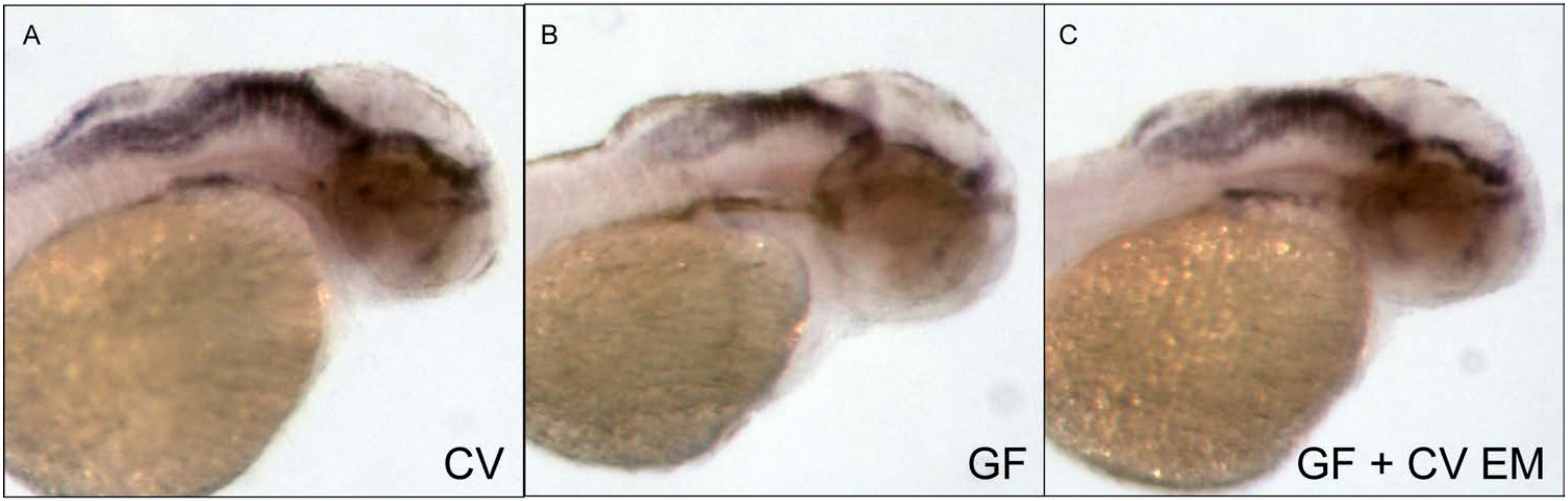

Supplemental Figure 1. WMISH of notch1b in 2dpf embryos. A) Conventionally raised embryo B) Germ-free embryo C) Embryo derived germ-free and immediately reintroduced to the embryo medium (EM) from which they were taken. (N = 8 embryos per treatment group).

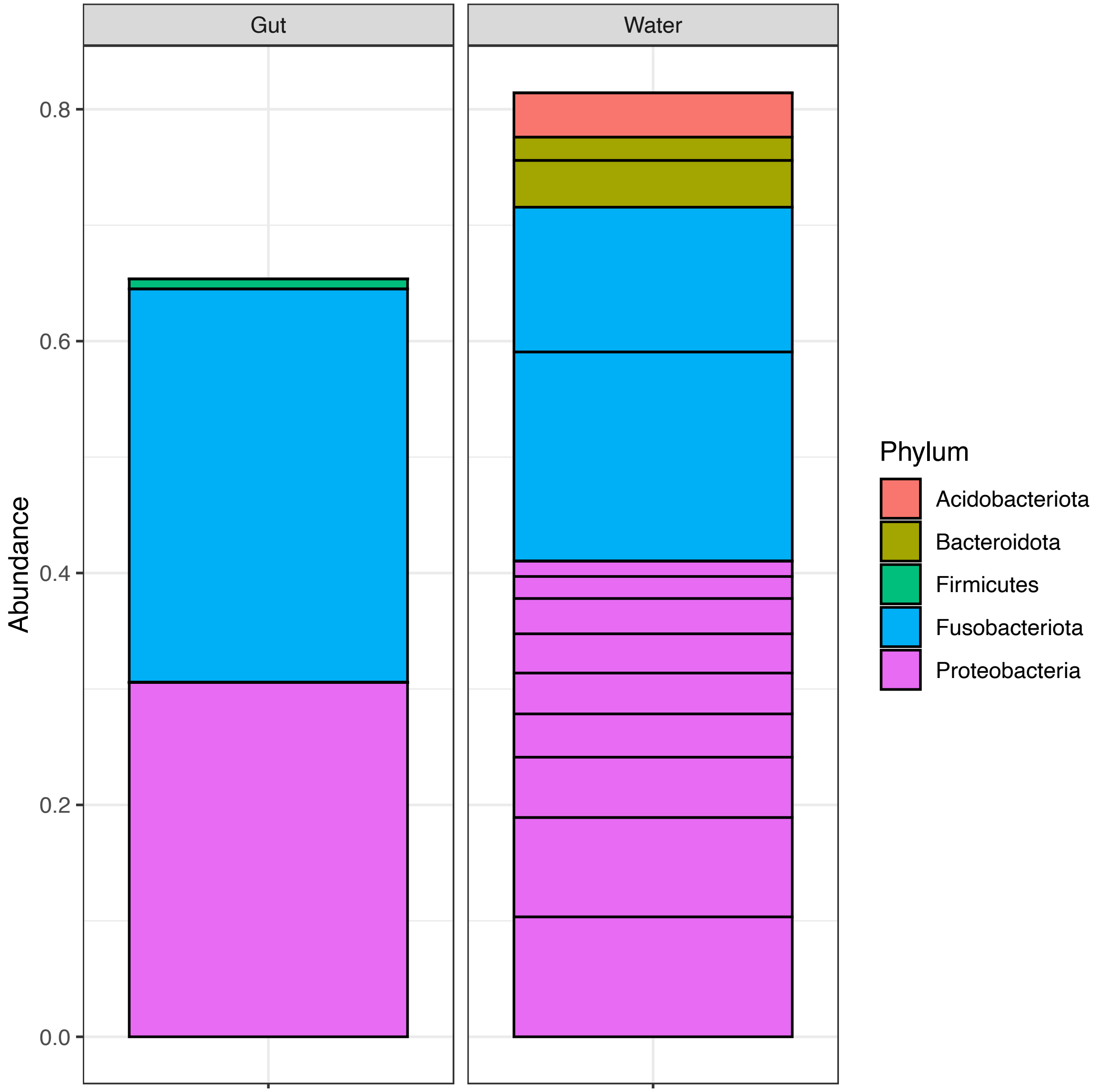

Supplemental Figure 2. Relative abundance of bacteria in zebrafish gut sample versus zebrafish water sample sample at the phylum level. Taxonomy was assigned using a training set of reference sequences via the Silva 138.1 prokaryotic SSU taxonomic training data formatted for DADA2.

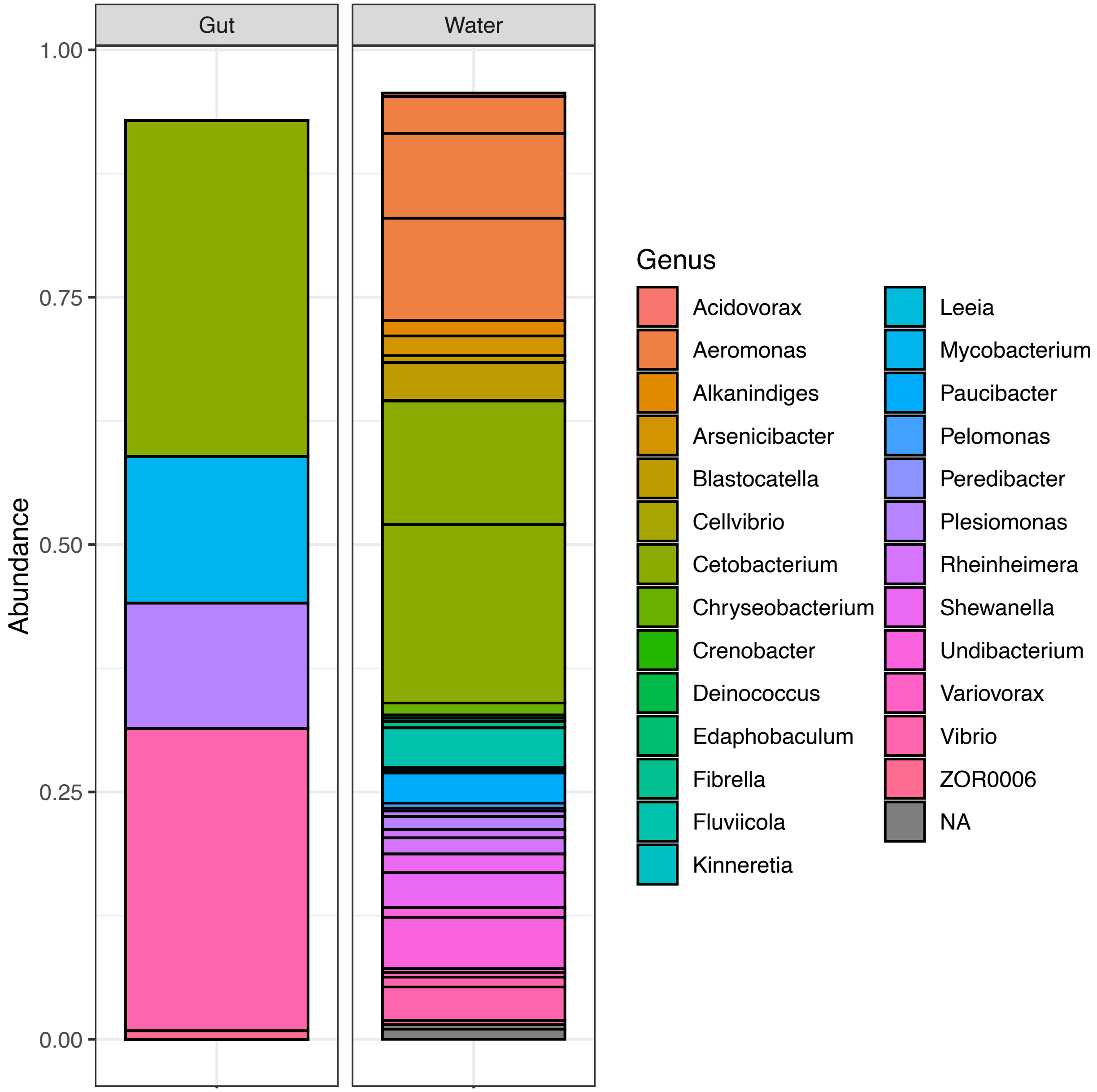

Supplemental Figure 3. Relative Abundance of bacteria in zebrafish gut sample versus zebrafish water sample at the genus level. Taxonomy was assigned using a training set of reference sequences via the Silva 138.1 prokaryotic SSU taxonomic training data formatted for DADA2.

axin2 - 2dpf

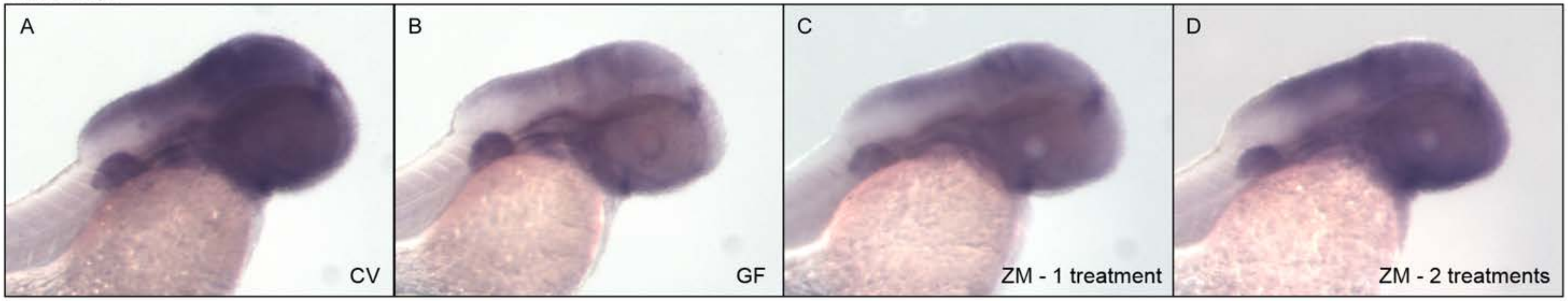

Supplemental Figure 4. WMISH of *axin2* in 2dpf A) conventionally raised embryos, B) germ-free embryos, C) germ-free embryos treated with zebrafish derived metabolites immediately after sterilization, and D) germ-free embryos treated with metabolites both immediately after sterilization and then again the next day. Zebrafish with two metabolite treatments have an increase in *axin2* expression compared to zebrafish with only one metabolite treatment. A total of 8 embryos were used in each group.

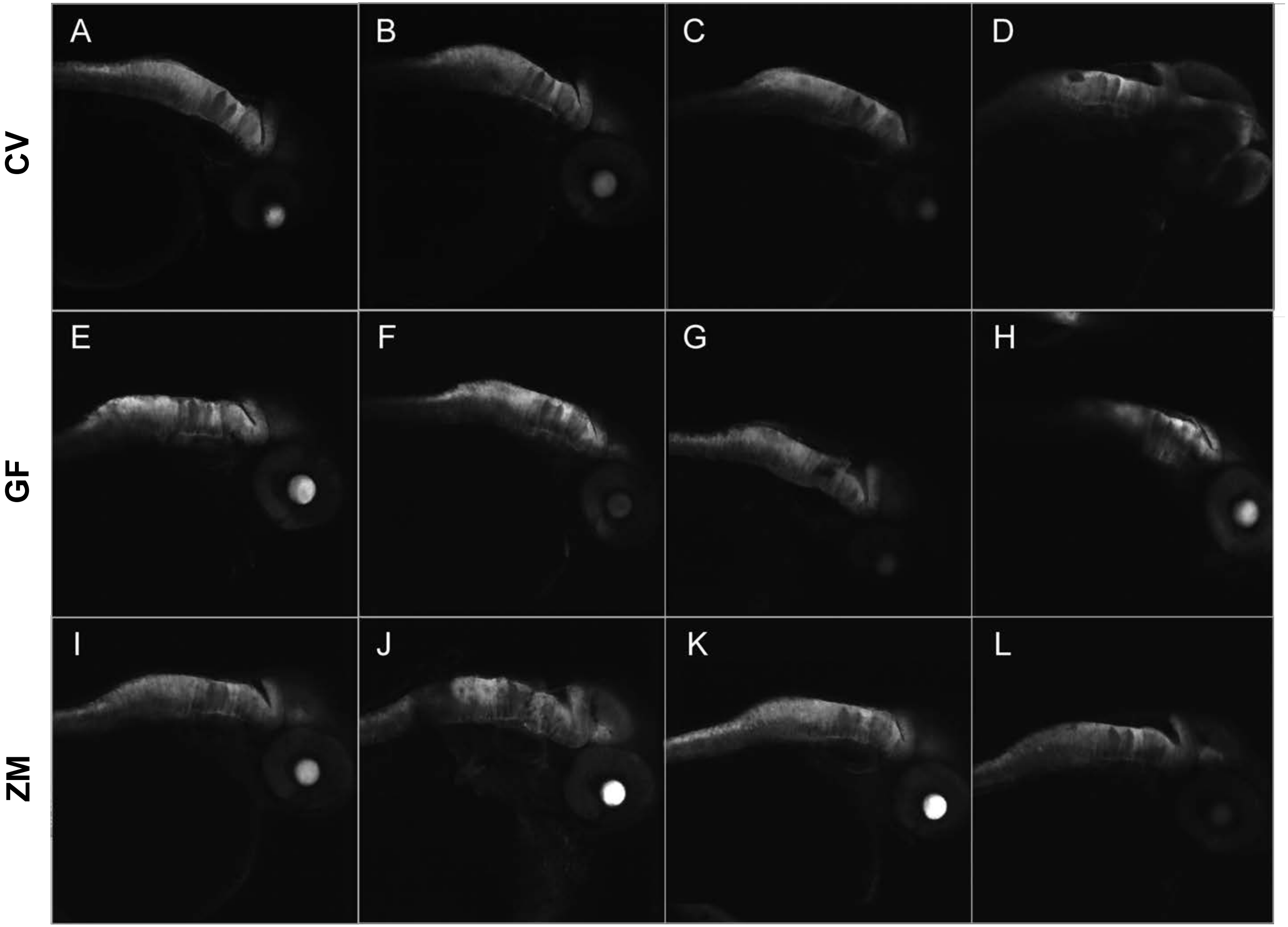

Supplemental Figure 5. Single layer composite using the Axonal tracts in the hindbrain as a guide. A-D) Conventionally raised embryos E-H) Germ-free embryos I-L) Germ-free embryos treated with zebrafish metabolites. Each image represents a different individual embryo in each treatment group.

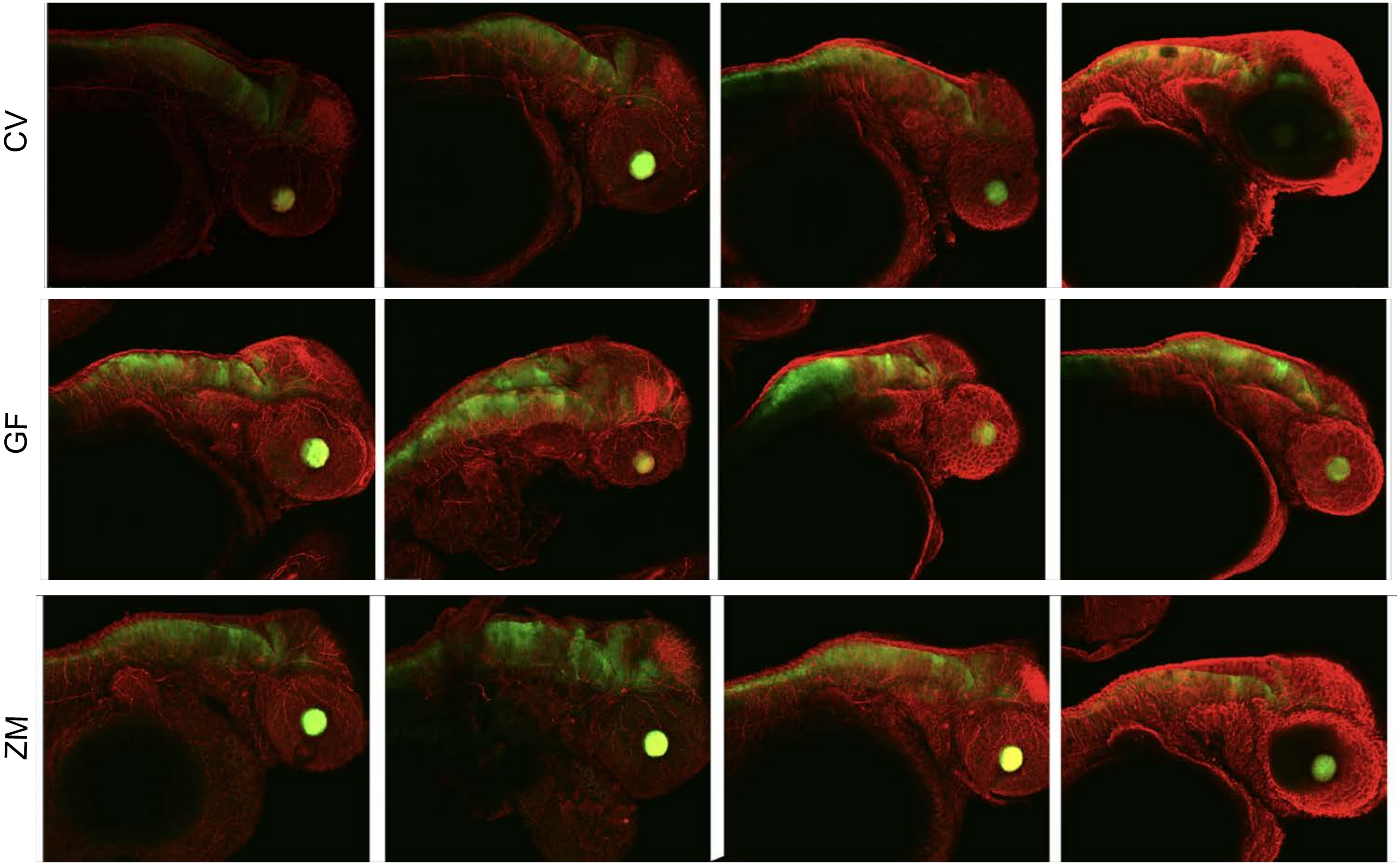

Supplemental Figure 6. Projected images of a-tubulin and GFAP-GFP expression. Each image represents a different individual embryo in each treatment group.

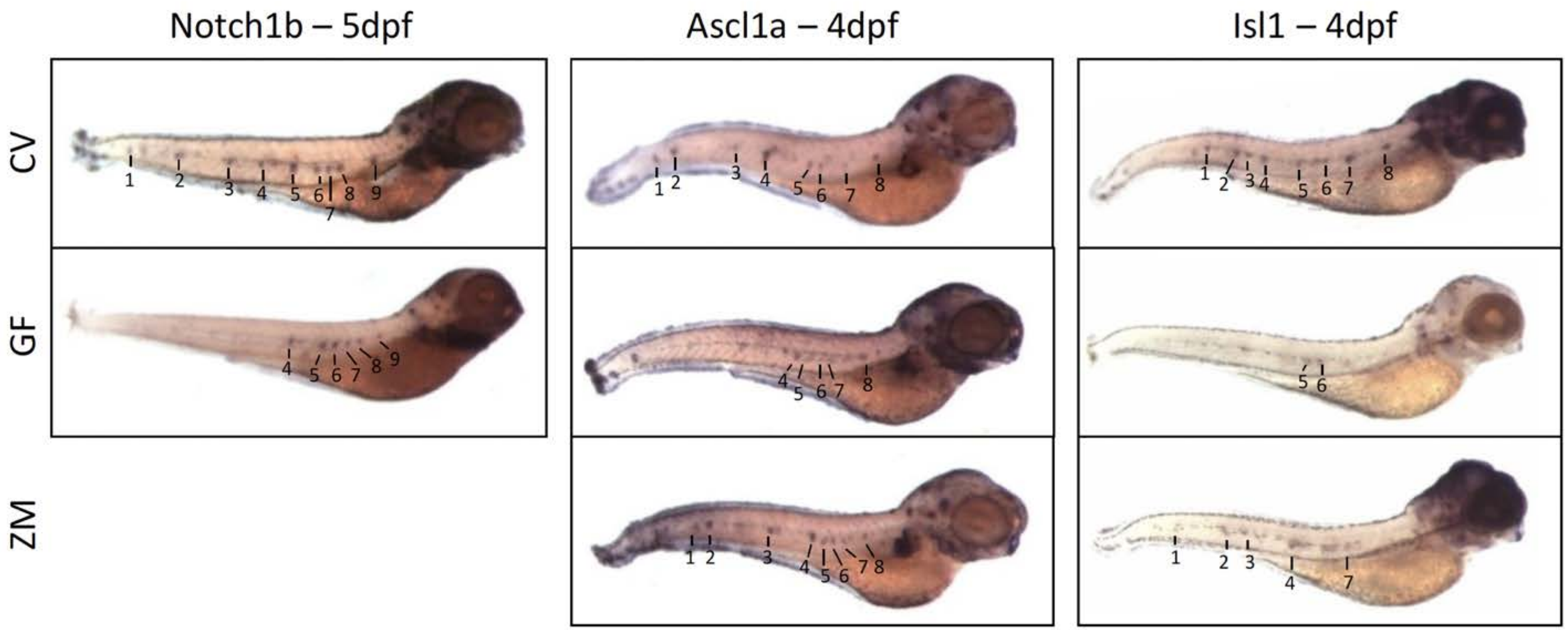

Supplemental Figure 7. Representative images of whole mount in situ hybridization on 5 dpf conventional larvae and germ-free larvae with Notch1b and 4dpf CV, GF and ZM larvae with acsl1a and Isl1. Lines and numbers identify neuromasts.

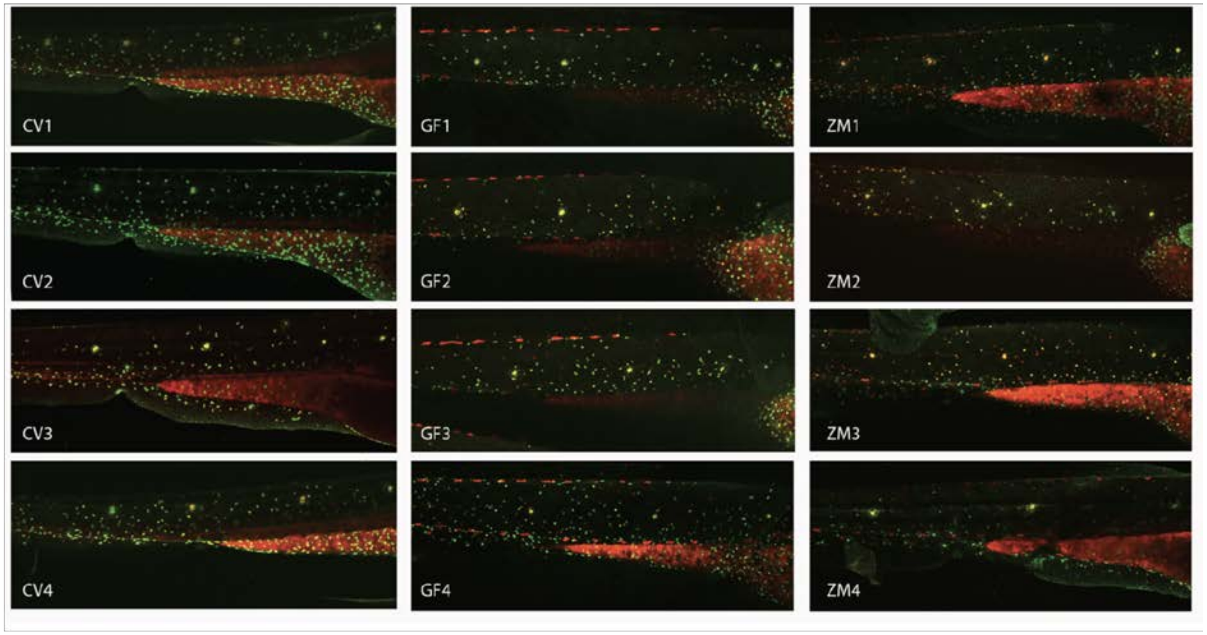

Supplemental Figure 8. Composite images of 3dpf larvae incubated in a mixture of Diasp and DioC6 to mark neuromast hair and accessory cells of the posterior lateral line. Different numbers represent different individual larvae of the same group.

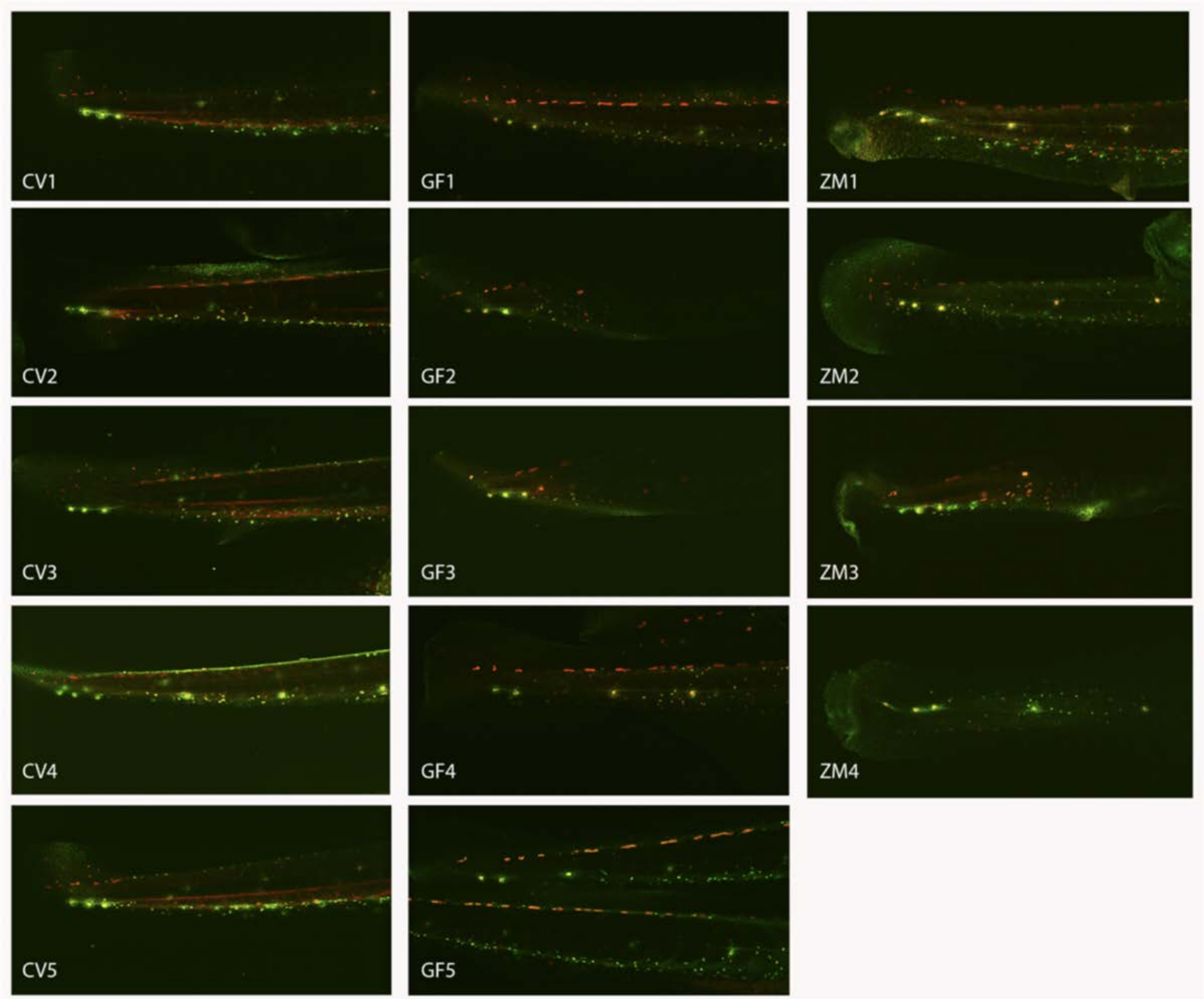

Supplemental Figure 9. Composite images of 3dpf larvae incubated in a mixture of Diasp and DioC6 to mark neuromast hair and accessory cells of the posterior lateral line. Images show the tail bud, with terminal neuromasts and posterior neuromasts of the trunk. Different numbers represent different individual larvae of the same group.

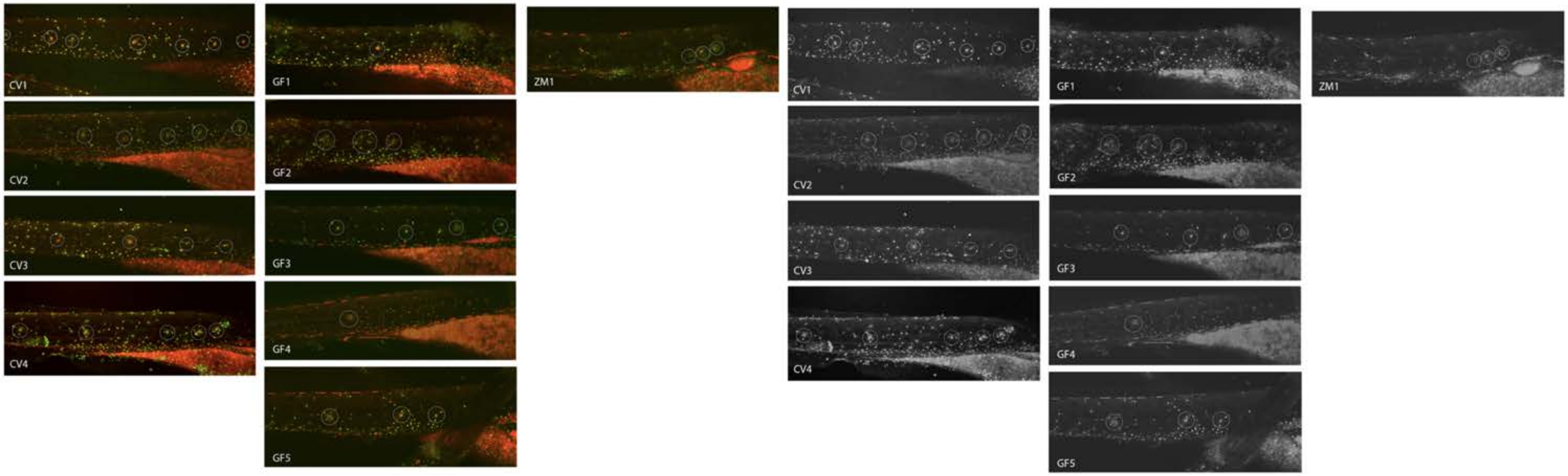

Supplemental Figure 10. Composite images of 4dpf larvae in a mixture of Diasp and DioC6 to mark neuromast hair and accessory cells of the posterior lateral line. The CV larvae have an average of 5 neuromasts (range from 4-7), whereas GF larvae [illegible] less on average (range from 1-4). Right image is composed of same images as left but in greyscale

Signalling pathways:

A

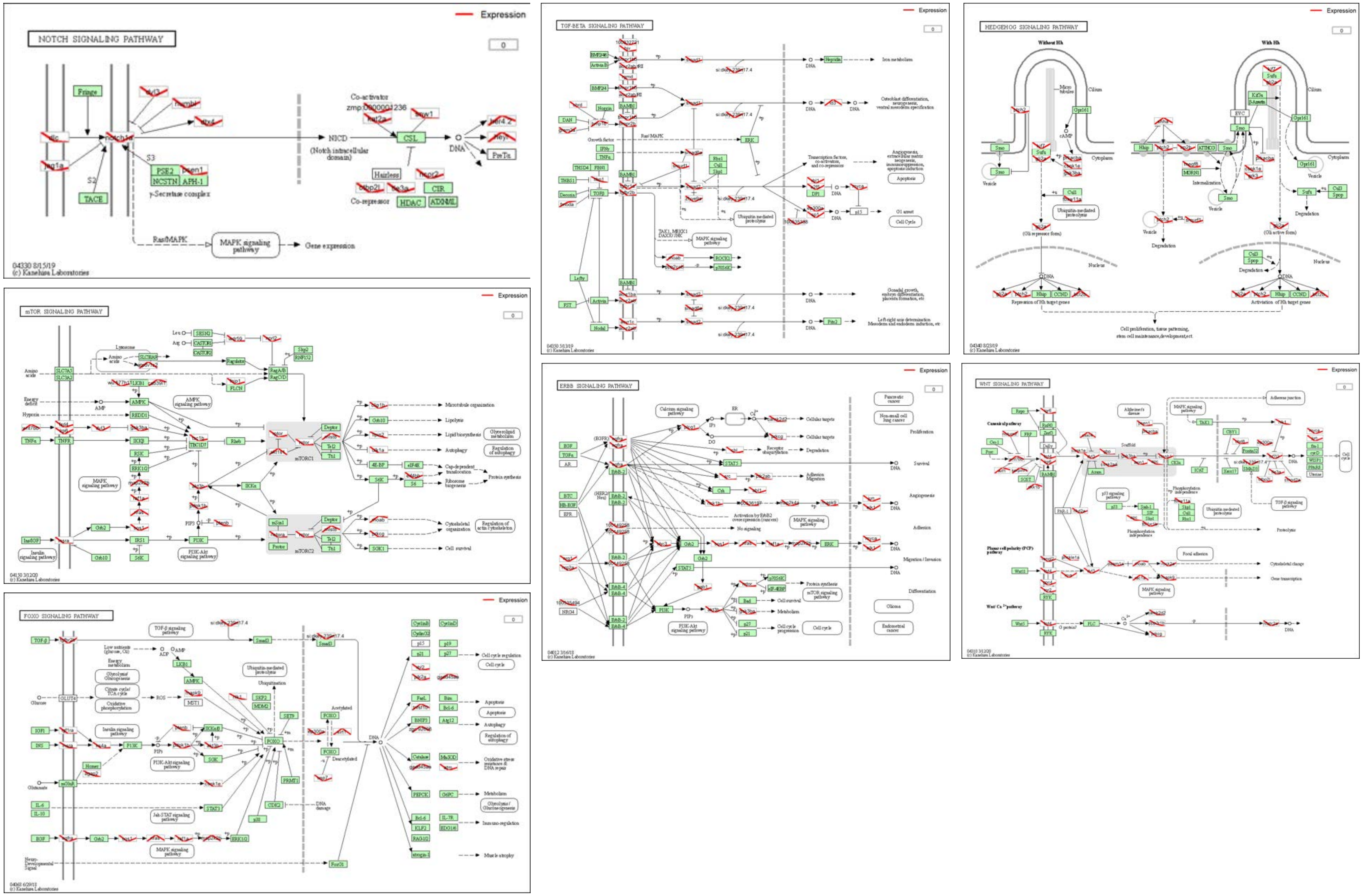

RNA associated:

B

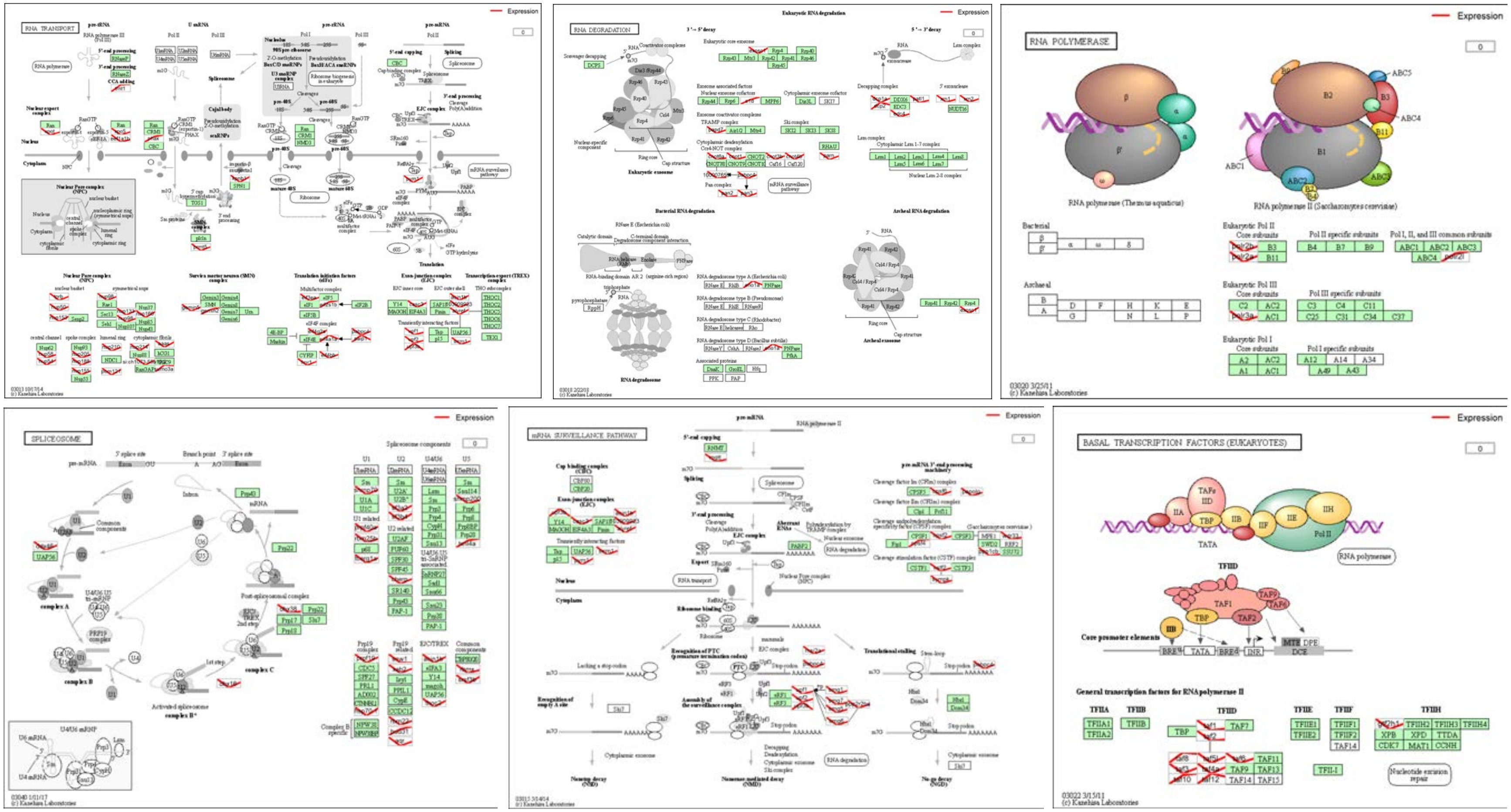

Other:

C

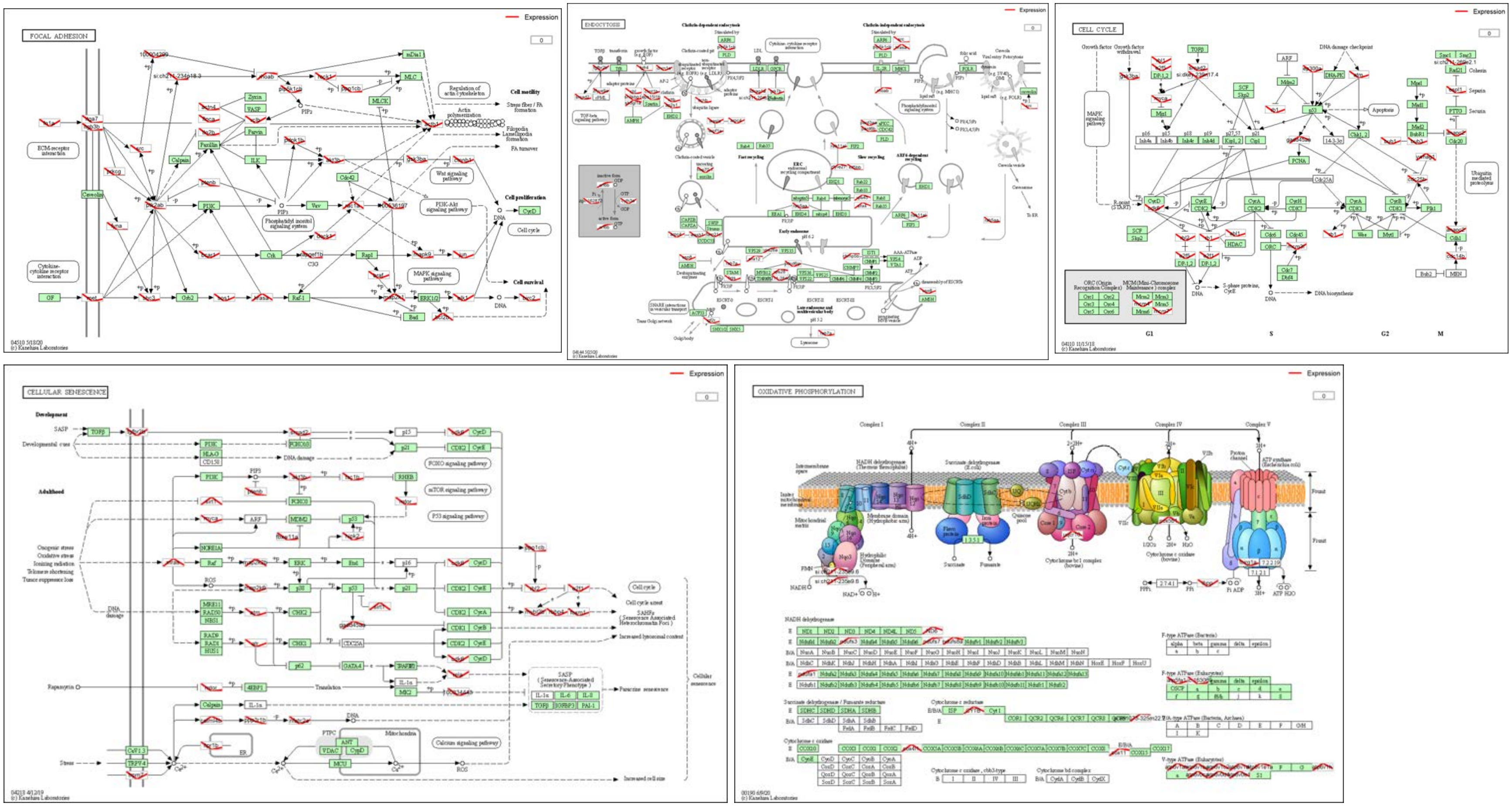

Supplemental Figure 11. KEGG profile output of important pathways and genes from complete RNA-Seq dataset. A) Signalling = pathways, B) RNA associated pathways and ontology, and C) other. Levels are shown in red where the left side (CV) is arbitrarily set to 0, the middle point is GF and the right point is ZM
